# Influence of Strong Molecular Vibrations on Decoherence of Molecular Polaritons

**DOI:** 10.1021/acsphotonics.4c01446

**Published:** 2024-11-15

**Authors:** Dominic M. Rouse, Erik M. Gauger, Brendon W. Lovett

**Affiliations:** † School of Physics and Astronomy, University of Glasgow, Glasgow G12 8QQ, U.K.; ‡ SUPA, Institute of Photonics and Quantum Sciences, Heriot-Watt University, Edinburgh EH14 4AS, U.K.; § SUPA, School of Physics and Astronomy, University of St Andrews, St Andrews KY16 9SS, U.K.

**Keywords:** dark states, decoherence, dephasing, master equation, molecules, polaritonics, polaron, transport

## Abstract

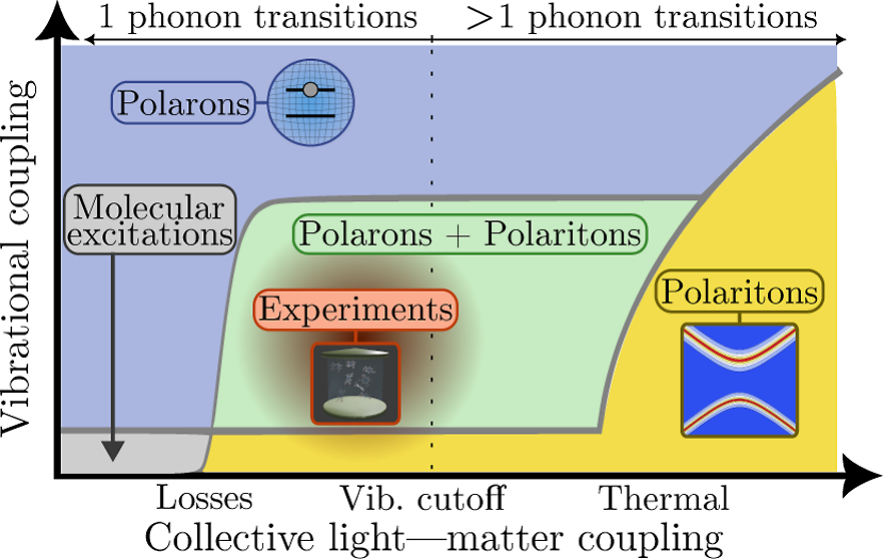

We derive the transition rates, dephasing rates, and Lamb shifts for a system consisting of many molecules collectively coupled to a resonant cavity mode. Using a variational polaron master equation, we show that strong vibrational interactions inherent to molecules give rise to multi-phonon processes and suppress the light–matter coupling. In the strong light–matter coupling limit, multiphonon contributions to the transition and dephasing rates strongly dominate over single-phonon contributions for typical molecular parameters. This leads to novel dependencies of the rates and spectral line widths on the number of molecules in the cavity. We also find that vibrational Lamb shifts can substantially modify the polariton energies in the strong light–matter coupling limit.

## Introduction

The confinement of a mode in a microcavity enhances the interaction strength between the light and charged matter within the cavity.^1^ The collective interaction strength also scales as the square root of the number of matter systems coupled to the light due to a collective enhancement.^2^ When the collective interaction strength exceeds dissipation mechanisms and disorder in the joint cavity–matter system, the cavity photons hybridize both in and out of phase with a symmetric superposition of the matter states—the so-called bright state—to form two polariton states. Polaritons inherit properties from both constituents, for example, a small effective mass from the photonic component, while retaining the material ability to interact with other polaritons.^3^ Additionally, since the bright state is delocalized across many matter systems—often tens-to-thousands of billions—polaritons also afford long-range control of matter properties.^4^ The remaining matter state superpositions—known as the dark states—remain uncoupled from the light mode.

Originally demonstrated in an atomic system,^5^ research has recently been directed toward using polaritonic physics to modify and control the properties of molecular systems, for example, superabsorption,^6^ energy transfer,^7,8^ chemical reactivities,^9−14^ photophysical dynamics,^15^ photoluminescence,^16^ and tunable coherence time scales.^17^ Molecular eigenstates are hybridizations of electronic excitations (excitons) with vibrational states that interact, often strongly, through displacement interactions. The joint excitonic–vibrational eigenstates are called polarons.^18^

The current state-of-the-art in analytical theoretical modeling of molecular polaritons is within the assumption of weak vibrational coupling,^19,20^ or for strong vibrational coupling but a single matter system.^21^ There has also been numerical calculations for an infinite number of matter systems using mean-field theory,^22^ to obtain steady-state properties and decay rates using the transfer tensor method,^23^ and in models with more complicated molecular descriptions such as with intramolecular coupling^24^ or including both triplet and singlet states.^25^

Weak vibrational couplings induce single-phonon transitions between all eigenstates of the joint cavity–molecule system, with the dark states acting as population traps when there are a large number of molecules.^19,20^ When the collective light–matter interaction strength exceeds the high-frequency cutoff of the vibrational baths—a regime now reachable in experiments^26^—single-phonon transitions between eigenstates are strongly suppressed. References (19 and 20) identify a zero frequency, single-phonon contribution to the polariton–ground-state decoherence which is suppressed only inversely with the number of molecules. Consequently, this contribution is purported to dominate the polariton line widths in the spectra when the collective light–matter coupling is strong.

On the other hand, when the vibrational couplings are strong, the collective light–matter couplings between the polarons and light mode are suppressed, and transitions between the eigenstates may also occur through multi-phonon processes.^21^ In practice, strong light–matter coupling in molecular cavity experiments is achieved with *N* ∼ [10^10^, 10^12^] molecules,^6,26^ resulting in two polariton states and *N* – 1 ≫ 2 dark states. The role of dark states in the strong vibrational coupling regime remains an open question because for the *N* = 1 system in ref (21), there are no dark states.

A polaron is a quasiparticle consisting of the molecular excitation dressed by the displaced vibrational modes coupled to the excitation.^27,28^ While a weak coupling master equation describes the dynamics of the molecular excitations, a polaron master equation describes the dynamics of the polarons which, crucially, does not break down at strong vibrational coupling. Broadly speaking, compared to a weak coupling master equation, a polaron master equation captures two key phenomena:^21^ screening of the light–matter coupling experienced by the excitation due to its phonon dressing and multiphonon processes due to the strong coupling.

Polaron master equations are perturbative in any coherent driving present in the system.^27^ In molecular polaritonics, the collective light–matter coupling is a coherent drive.^21^ This poses a problem for polaron physics because polariton formation is synonymous with strong light–matter coupling. However, a variational polaron master equation (VPME) is well-known to overcome this hurdle—e.g., in the spin boson model^29^—by varying the magnitude and mode structure of the phonon dressing as a function of the coherent drive strength relative to the vibrational coupling strength.

**Figure 1:**
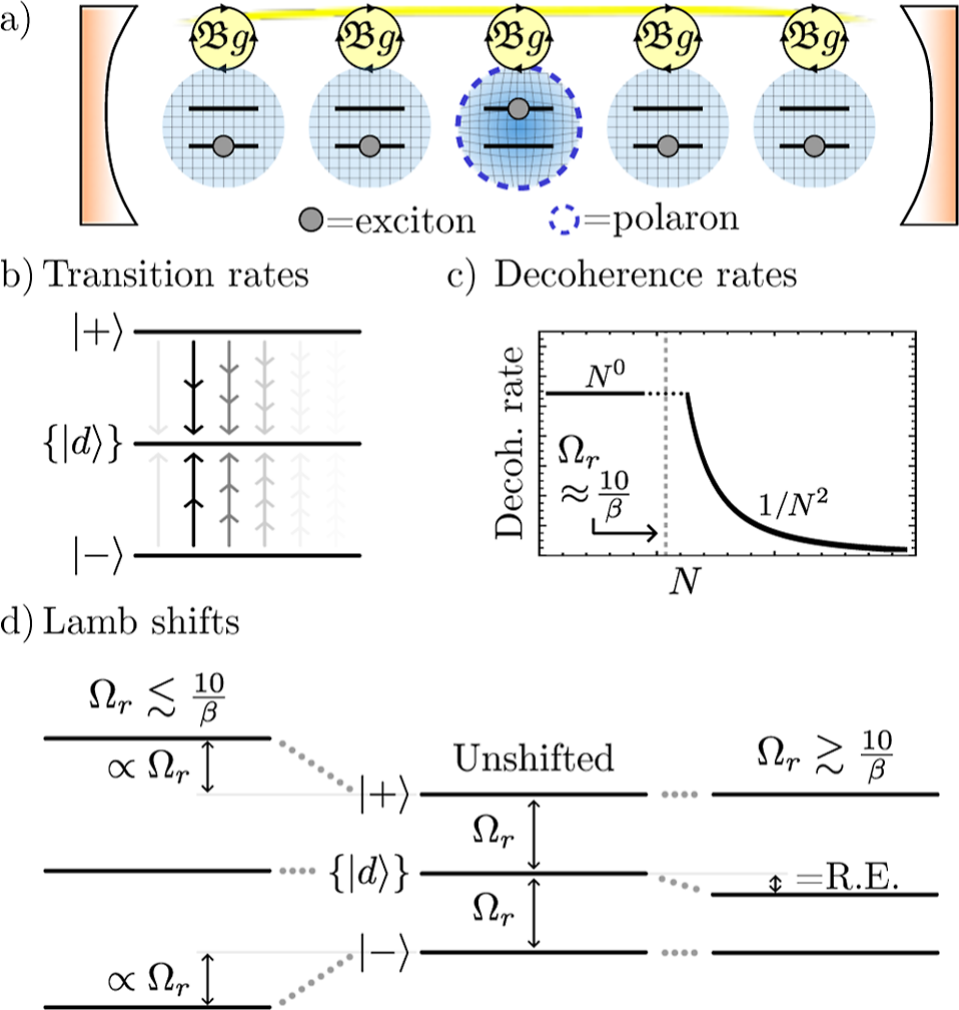
Illustration of our model and main results. (a) Our model consists of *N* two-level systems, each coupled to a common cavity mode which causes polariton formation, and to a local vibrational bath which causes polaron formation. The phonon dressing within the polaron suppresses the light–matter coupling strength *g* by a factor 0 <  < 1. In the large *N* limit, our main results are as follows. (b) The *N* – 1 degenerate dark states {|*d*⟩} act as population traps (in agreement with refs (19 and 20)) and, if , where ω_0_ is the high-frequency cutoff of the vibrational baths, then multi-phonon processes strongly dominate over single-phonon processes. Typically, processes with 2 or 3 phonons dominate, indicated by the saturation of the arrow colors. (c) Decoherence rates have different *N* scaling depending on the size of the collective light–matter coupling Ω_*r*_ compared to the inverse temperature β. When Ω_*r*_ ≲ 10/β, decoherence is dominated by processes with even numbers of phonons, while when Ω_*r*_ ≳ 10/β, *only* two-phonon processes contribute significantly. When Ω_*r*_ ∼ Ω_β_, the *N* dependence is more complicated and not illustrated. (d) When Ω_*r*_ ≲ 10/β, the polariton states are Lamb shifted by an equal and opposite amount proportional to the bare splitting Ω_*r*_ which can be substantial for moderately strong vibrational coupling, while when Ω_*r*_ ≳ 10/β, only the dark states are shifted by the vibrational reorganization energy (“R.E.”). All Lamb shifts shown result from transitions involving the dark states. Typical molecular experiments operate within the regime Ω_*r*_ ≲ 10/β but can reach Ω_*r*_ ≳ ω_0_.^6,26^

In this paper, we derive analytical expressions for the key dynamical rates and Lamb shifts that remain accurate when the vibrational coupling is strong and for an arbitrary number of molecules in the cavity. We analyze our expressions for the parameter regimes most relevant to recent experiments.^6,26^ Our methodology builds upon refs (19 and 20) by utilizing a variational polaron transformation and upon ref (21) by including an arbitrary number of molecules. Our model and main results are illustrated in Figure 1.

This article is organized as follows. After introducing the Hamiltonian of the model, we summarize refs (19 and 20) by deriving the transition rates, decoherence rates, and Lamb shifts valid when the vibrational couplings are weak. Following this, we transform the Hamiltonian into the variational polaron frame and identify parameter regimes with distinct dependencies on the light–matter and vibrational couplings. We also show that when the light–matter and vibrational couplings are simultaneously strong, “resonant” cavity experiments should be modeled by nonresonant Hamiltonians. We then study the expressions for transition rates, decoherence rates, and Lamb shifts in the VPME with an emphasis on presenting simple and generic conclusions for the parameter regime most relevant to experiments. Finally, we discuss corrections for nonresonant systems.

## Model

We consider *N* identical molecules treated as two-level systems with transition energy ω_m_ and independent but identical vibrational baths, see Figure 1a. The transition dipoles of the molecules couple to the cavity mode of energy ω_c_ with a light–matter coupling of *g*. We neglect permanent dipole interactions with the cavity which is a typical assumption within molecular polaritonics.^13^ This is the localized bath model considered in refs (19 and 20).

We partition the Hamiltonian into *H* = *H*_S_ + *H*_B_ + *H*_SB_. The system, which consists of the quantized cavity mode and molecules, is described by
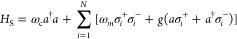
1where *a*^†^ and σ_*i*_^+^ create an excitation in the cavity mode and *i*th molecule, respectively. Later we will enforce resonance between the cavity mode and the molecular transition. However, the requirement for resonance differs for weak and strong vibrational couplings, and so we discuss each in the appropriate sections.

The vibrational baths of the molecules are described by
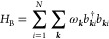
2where *b*_***k****i*_^†^ creates a phonon of wavevector ***k*** and energy ω_***k***_ in the vibrational bath of the *i*th molecule. The displacement interactions induced by the vibrational baths are described by
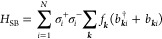
3where *f*_***k***_ is the coupling strength of a mode with wavevector ***k***. The vibrational coupling of each molecule to its local bath is characterized by the spectral density, *J*(ω) = ∑_***k***_*f*_***k***_^2^δ(ω – ω_***k***_). We consider spectral densities of the form
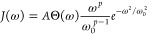
4where ω_0_ is the high-frequency cutoff, *p* is the Ohmicity, Θ(ω) is the Heaviside step function, and *A* is a dimensionless coupling constant with *A* ≳ 0.1 signaling strong coupling. We choose eq 4 to make connection with refs (19 and 20) where the Ohmic (*p* = 1) case is studied at weak vibrational coupling, although our results hold for any spectral density which does not suffer from the well-known infrared divergence of the variational polaron transformation.^30,31^Equation 4 is suitable for modeling the broad low-frequency contribution to molecular spectral densities^32^ but omits the peaked structure that can generate strongly non-Markovian dynamics.^33−35^

Realistic values for the model parameters can be identified from recent experiments.^6,26^ In refs (6 and 26,) phonon renormalized light–matter couplings of  and  = 0.1 μeV and maximum collective light–matter coupling strengths  of 4.2 and 20.7 meV were found, respectively. As demonstrated in Supporting Information Section S1, we estimate *A* = 0.083 for the dye molecules in ref (6) which is near to the strong vibrational coupling regime. In ref (26), the cutoff frequency for the bath is ω_0_ = 6 meV, and the experiments in both refs (6 and 26) were performed at room temperature *T* = 1/β = 0.0258 eV which is typical in molecular polaritonics. We will frequently refer to *typical molecular parameters* which we take as the following: bare light–matter coupling *g* = 0.1 μeV, high-frequency cutoff ω_0_ = 6 meV, vibrational coupling strength *A* = 0.083, and temperature *T* = 300 K.

We have chosen to model the light–matter coupling as between the transition dipoles of the molecules and the cavity—resulting in a two-level system description of the excitation, as also chosen in ref (21)—rather than between the vibrational excitations and the cavity—resulting in a bosonic description of the excitation, as chosen in refs (19, 20, and 24). Both models yield the same results in the single excitation manifold which, following refs (19 and 20) and motivated in the next section, we work within.

## Weak Vibrational Coupling Master Equation

In this section, we summarize the results of refs (19 and 20) relevant to our study by deriving the Redfield master equation resulting from *H*_SB_ in eq 3 perturbing *H*_S_ + *H*_B_ in eqs 1 and 2. This weak vibrational coupling master equation (WCME) becomes inaccurate once the vibrational and collective light–matter couplings become comparable. We will introduce a parameter to quantify this comparison when we introduce the variational polaron transformation.

**Figure 2:**
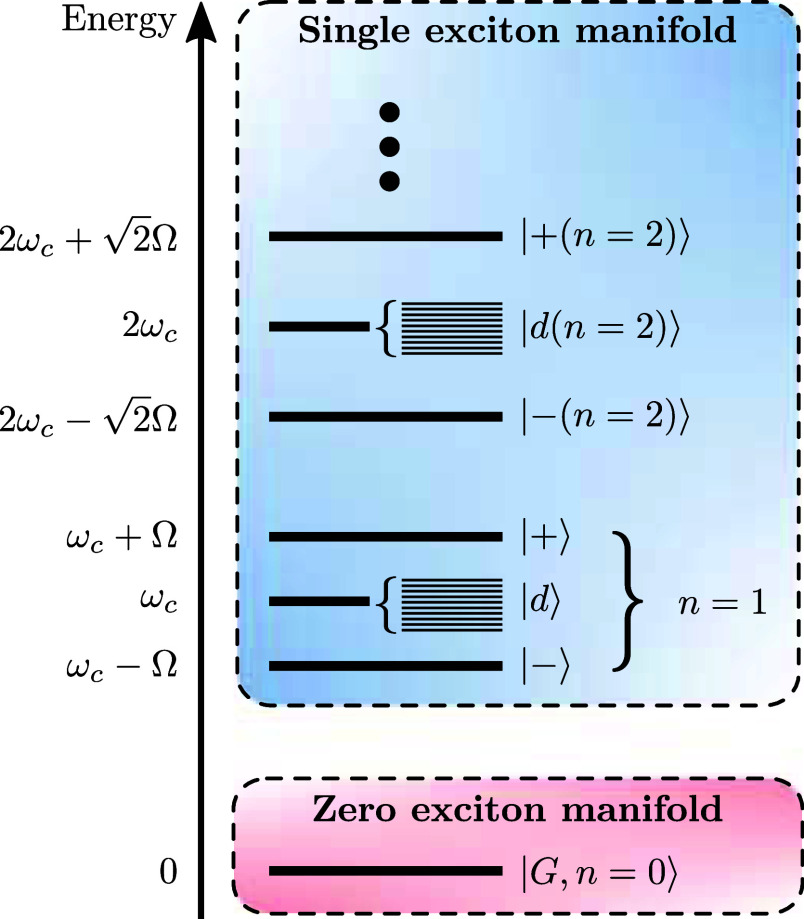
Zero and single exciton manifolds of *H*_S_ when the cavity mode is resonant with the molecular transition energy. Due to the symmetry of *H*_S_, each set of *N* + 1 states identified by photon number *n* ≥ 1 are decoupled. To study the linear response of the system, we study the dynamics for *n* = 1.

Deriving the master equation requires diagonalizing *H*_S_ in eq 1. We assume that the total number of excitons at any given time does not exceed one,^19^ which, because *H*_S_ preserves the total number of excitations, decouples the eigenstates into sets uniquely identified by photon number *n*. Each set is spanned by *N* + 1 states, {|*G*, *n*⟩, |*e*_*i*_, *n* – 1⟩_*i*=1,···,*N*_}, where |mol, *n*⟩ = |mol⟩⊗|*n*⟩ is a product state with *n* photons and either zero excitons (|mol⟩ = |*G*⟩) or an exciton in the *i*th molecular state only (|mol⟩ = |*e*_*i*_⟩). The set relevant to the master equation is determined by the number of photons in the cavity, which we assume to be constant on time scales induced by the vibrational interactions. Following ref (19), we choose *n* = 1, but, as shown in Figure 2, the states with *n* > 1 differ only by constant factors in the transition energies (the transition energies between the eigenstates increase when there are more photons in the cavity. As discussed later, this will change whether single-phonon or multi-phonon processes dominate the transition rates).

In the recent experiments discussed previously, the photon leakage rates are 5.5^6^ and 17 meV,^26^ which are about three and five times *faster* than the respective vibrational decoherence rates. Evidently, an assumption that the photon number is constant during the dynamics is not always accurate. However, photon leakage—causing transitions between constant excitation number manifolds—and vibrational interactions—causing transitions within these manifolds—are largely independent of one another. So we can neglect cavity leakage to keep the analysis focused on the vibrational physics. In Supporting Information Section S2, we show the effects of cavity leakage (and nonradiative molecular decay) on our main results.

The *n* = 1 single excitation subspace of *H*_*S*_ contains an upper polariton | + ⟩, lower polariton | – ⟩, and *N* – 1 degenerate dark states {|*d*⟩} for *d* ∈ {*d*_1_...*d*_*N*–1_}. The upper and lower polaritons are symmetric and antisymmetric superpositions of single excitation states
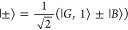
5where  is the bright state. The dark states
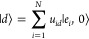
6are the *N* – 1 degenerate superpositions of single exciton states orthonormal to |*B*⟩. The coefficients *u*_*id*_ are complex valued and satisfy
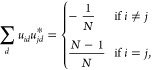
7
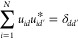
8
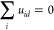
9which enforces unit trace of the density operator (eq 7) and orthonormality of the eigenstates (eqs 8 and 9).

Resonance between the cavity mode and molecular transitions in eq 1 is enforced by choosing ω_*c*_ = ω_*m*_. On resonance, the polariton energies are ω_±_ = ω_*c*_ ± Ω where
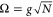
10is the collective light–matter coupling, and, regardless of resonance, the dark states have an energy ω_*d*_ = ω_*m*_.

Denoting the reduced density operator for the light–matter system as ρ_S_(*t*) = Tr_B_[ρ(*t*)]—where Tr_B_[·] is the trace over the joint Hilbert spaces of the vibrational baths—one finds that the WCME in the Schrödinger picture^36^ is
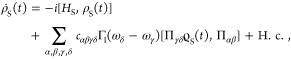
11where Π_αβ_ = |α⟩⟨β| is an eigenstate transition operator, ω_δ_ is the energy of eigenstate |δ⟩, Greek indices sum over all eigenstates in the *n* = 1 single exciton manifold, and “H.c.” denotes the Hermitian conjugate. The coefficients are
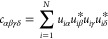
12where *u*_*i*α_ = ⟨*e*_*i*_, 0|α⟩ such that  and *u*_*id*_ satisfy eqs 7–9. The Fourier transform of the bath correlation function is
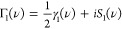
13where
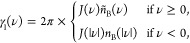
14with *n*_B_(ν) = 1/(exp(βν) – 1), the Bose–Einstein distribution, , and
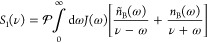
15where  denotes the principal value.

The subscript ‘1’ denotes that the correlation function originates from single-phonon interactions. Equation 11 is the same nonsecular master equation, as derived in refs (19 and 20). In this paper, we are interested only in the transition rates, dephasing rates, and Lamb shifts of the eigenstates, which are secular contributions to the master equation. These terms can be obtained from any master equation by deriving the coefficient of the element ρ_μν_(*t*) ≡ ⟨μ|ρ_*S*_(*t*)|ν⟩ within the equation of motion for , where μ and ν label any eigenstates of *H*_S_. This term takes the general form
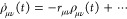
16where
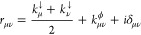
17and terms in the ellipsis in eq 16 do not depend on ρ_μν_(*t*). In 17 we have defined the total loss rate of state |μ⟩ as
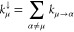
18where *k*_μ→α_ is the transition rate from state |μ⟩ to |α⟩. We have also defined the dephasing rate, *k*_μν_^ϕ^, of the coherence between states |μ⟩ and |ν⟩ with the properties *k*_μμ_^ϕ^ = 0 and *k*_μν_^ϕ^ = *k*_νμ_^ϕ^. The last term in eq 17 is the Lamb shifted transition energy from state |μ⟩ to |ν⟩, given by
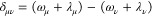
19where λ_μ_ is the Lamb shift of state |μ⟩. In the following subsections, we analyze the expressions for the quantities appearing in eq 17.

### Transition Rates

The transition rates obtained from the WCME in eq 11 are
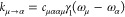
20

Evaluating *c*_μααμ_ using eq 12, we find
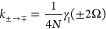
21
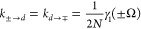
22
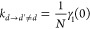
23and all transition rates involving the zero excitation state |*G*, 0⟩ are zero. Equations 21 and 22 describe decay by single-phonon emission (positive-frequency arguments) and excitation by single-phonon absorption (negative-frequency arguments). These expressions take the form of Fermi’s Golden Rule. Equation 23 describes transitions between degenerate states, which, being zero-frequency transitions, do not contribute to overall population transfer. However, these transitions contribute to decoherence and Lamb shifts.

From eqs 21–23, we obtain the following loss rates for each state
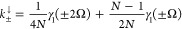
24
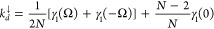
25

As noted by ref (19), since there are (*N* – 1)/2 times more dark states than polaritons, in the large *N* limit the dark states act as population traps.

### Dephasing Rates

The dephasing rates obtained from the WCME are
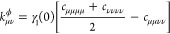
26

We now briefly introduce terminology to distinguish three possible contributions to decoherence. Generally, the decoherence rate of the coherence between states |μ⟩ and |ν⟩ is the real part of *r*_μν_ in eq 17 for μ ≠ ν. The three possible contributions to this are (1) transitions from either state, (2) virtual transitions from either state back to itself, or (3) other pure dephasing contributions. While transitions contribute to decoherence, they are not dephasing processes. The first term in eq 26 arises from virtual transitions from |μ⟩ → |μ⟩ and |ν⟩ → |ν⟩, and the second term from other pure dephasing contributions. Both dephasing processes depend on the zero-frequency bath correlation function.

Using eq 12 to evaluate *c*_μμμμ_ and *c*_μμνν_, we find the following dephasing rates
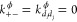
27
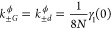
28
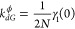
29where subscript “*G*” refers to the zero excitation state |*G*, 0⟩. The dephasing rate of the coherence between |±⟩ and |∓⟩, denoted *k*_+–_^ϕ^, is zero because only the molecular parts of the polariton wave functions interact with the vibrational baths—see *H*_SB_ in eq 3—and both polaritons feature the same molecular wave function up to a phase.^20^ This symmetry will be broken by the variational polaron transformation.

As shown in Supporting Information Section S3, within the validity of the WCME—where phonon sidebands are small—and of the quantum regression theorem—where the Born and Markov approximations hold^37^—the absorption spectrum of the cavity is given by
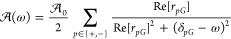
30where  is the intensity. The spectrum describes two Lorentzian peaks centered at the Lamb shifted polariton energies, with full width at half-maxima equal to 2Re[*r*_±*G*_] = *k*_±_^↓^ + 2*k*_±*G*_^ϕ^. As discussed in ref (19), eq 28 predicts that when *N* is large enough to satisfy Ω ≫ ω_0_—so that transition rates are exponentially suppressed by the high-frequency cutoff of the vibrations—the width of the polariton peaks in the cavity absorption spectrum will be dominated by *k*_±*G*_^ϕ^ in eq 28, which scales as 1/*N*. As illustrated in Figure 1c, this prediction will be challenged by the VPME.

The single-phonon dephasing rates in eqs 28 and 29 depend on
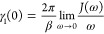
31

Equation 31 is only nonzero and nondivergent if the spectral density is exactly linear in frequency at small frequencies, which is unlikely to be the case in molecular systems. Generally, pure dephasing cannot have single-phonon Markovian contributions because it results from interactions between the system and the baths which do not lead to energy exchange. Therefore, single-phonon Markovian pure dephasing could only occur through emission, or, equivalently absorption, of a zero-frequency phonon, resulting in the spurious expression in eq 31 which varies discontinuously as the Ohmicity changes. Conversely, single-phonon *non*-Markovian pure dephasing is entirely possible—the system emits a finite energy phonon which is later reabsorbed—and so is *multi*-phonon Markovian pure dephasing—the system simultaneously emits and absorbs an equal number of phonons of the same finite frequency.

Single-phonon non-Markovian processes can be described by the WCME in eq 11 if the Markovian assumption in the bath correlation function is relaxed. However, multiphonon Markovian processes require a strong vibrational coupling theory. We defer the discussion of both processes so that the present section remains a faithful summary of refs (19 and 20).

### Lamb Shifts

The Lamb shifts obtained from the WCME are
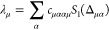
32where *S*_1_(ν) is given in eq 15. The frequency dependence of *S*_1_(ν) is generally complicated, but, when |ν|≫ ω_0_, one can ignore the ±ω in the denominators of the integrand in eq 15, resulting in *S*_1_(ν) ∼ 1/ν for any spectral density. Consequently, when Ω ≫ ω_0_, Lamb shifts resulting from terms with μ ≠ α in eq 32 scale as *c*_μααμ_/Ω which will be negligible in comparison to the bare energy splittings, scaling as Ω, when *N* is large. Additionally, since , the only Lamb shifts with μ = α that may be comparable to the bare energy splitting at large *N* are the contributions to λ_*d*_ resulting from real and virtual dark state transitions. One finds that these contributions are
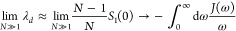
33which is the negative of the reorganization energy of the vibrational baths. Therefore, if the vibrational coupling is comparable to the Rabi frequency, |*S*_1_(0)| ≳ Ω, then the dark state energy may be non-negligibly Lamb shifted. However, in this regime, the WCME is no longer accurate.

### Summary of Weak Vibrational Coupling

We have derived the Redfield equation in the limit of weak vibrational coupling and recovered the same expressions for the transition rates, decoherence rates, and Lamb shifts found in refs (19 and 20). There remain a number of questions unanswered by weak vibrational coupling theory.

(1) For the single matter system model in ref (21), strong vibrational coupling suppressed the bare light–matter coupling strength and introduced multi-phonon processes. How does the suppression scale with the number of molecules? Do multi-phonon processes qualitatively change the dynamics?
(2) When Ω ≫ ω_0_, the WCME predicts that the decoherence rates—and so the line widths of the polaritons—are dominated by the 1/*N* pure dephasing contribution from the single-phonon Markovian process described by γ_1_(0) given in eq 31. This term produces divergences or zero values for most spectral density types. What happens if the Markovian assumption is relaxed? To leading order, is decoherence a multi-phonon process?
(3) Are the Lamb shifts still negligible in the large *N* limit when the vibrational coupling is strong?

The answer to these questions requires strong vibrational coupling theory.

## Variational Polaron Theory

A polaron master equation is a Redfield equation derived after transforming the Hamiltonian *H* by state-dependent phonon displacement. The resulting master equation is perturbative in a quantity that remains small when the vibrational coupling is strong.^27^ However, the perturbative quantity grows proportional to any driving in the system, which, in our model, is the collective light–matter coupling. This breakdown can be mitigated by employing a variational version of the unitary transformation.^29,38,39^ The unitary operator is optimized such that the Gibbs state of the unperturbed Hamiltonian is as close to the equilibrium steady state as permitted by a polaron-type transformation, and so the residual interaction is more amenable to perturbation theory.

The Hamiltonian in the variational frame is  where
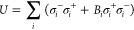
34and *B*_*i*_ = exp[−∑_***k***_(η_***k***_/ω_***k***_)(*b*_***k****i*_^†^ – *b*_***k****i*_)] is a displacement operator. We use calligraphic notation to denote operators transformed into the variational frame. Equation 34 describes a transformation that displaces a vibrational bath when the corresponding molecule is in its excited state but otherwise does not transform the bath. The variational parameters, η_**k**_, are free parameters used to optimize the transformation and have the general form η_***k***_ = *G*(ω_***k***_)*f*_***k***_ where
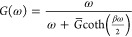
35The intuition within eq 35 is that, after molecular excitation, the low-frequency phonon modes displace more slowly than high frequency ones, with the boundary between slow and fast modes determined by the parameter *G̅*, which we will define soon.

After applying the transformation in eq 34, we find the polaron Hamiltonian . The system part is the Tavis–Cummings Hamiltonian with renormalized molecular energy and light–matter coupling
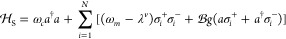
36where
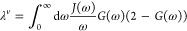
37and 0 <  < 1 is a suppression of the light–matter coupling by the vibrational coupling, given by
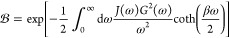
38the bath part of the Hamiltonian is the same as before the transformation, , and the interaction Hamiltonian has two components, . There is a displacement-type interaction
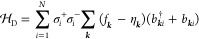
39named due to its similarity to eq 3, and a polaron-type interaction
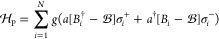
40when η_***k***_ → 0 the polaron-type interaction vanishes  and the Hamiltonian reverts back to its original form . Conversely, when η_***k***_ → *f*_***k***_ the displacement-type interaction vanishes  as the polaron incorporates the total energy of the displacement described by *H*_SB_. Generally, the η_***k***_ range from 0 to *f*_***k***_ as the frequency of the mode increases, such that both interaction types contribute to the dynamics with a weighting determined by *G*(ω).

Notice that we have redefined the partition between the system and interaction Hamiltonians, such that  for any α ∈ {S, B, SB}. This partition is made to ensure —where —so that perturbation theory in  yields the familiar Redfield equation.

In the weak vibrational coupling theory, resonance in *H*_S_ was enforced by choosing the cavity energy ω_*c*_ = ω_*m*_, where ω_*m*_ is the energy of the molecular excitation. Clearly, the same choice for ω_*c*_ in  in eq 36 does not yield a resonant Hamiltonian. Moreover, since the variational polaron frame molecular energy ω_*m*_ – λ^*v*^ depends on *G*(ω), the value of ω_*c*_ that leads to resonance implicitly depends on itself. This leads one to consider how to properly enforce resonance. The relevant question is, how, in the experiments we are modeling, is the molecular energy determined?

To answer this question, it is helpful to consider the Hamiltonian for an isolated molecule
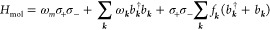
41*H*_mol_ is diagonalized by a full polaron transformation—the variational polaron transformation with *G*(ω) = 1—yielding , where *S*_1_(0) is given in eq 33. In a measurement of the molecular energy—for example, by coupling the molecule to a weak probe field and measuring the absorption spectrum—the vibrational reorganization energy *S*_1_(0) cannot be separated from the excitonic energy ω_*m*_. Therefore, after assuming that the Lamb shift induced by the weak probe field is negligible, one would attempt to enforce the resonance by building a cavity with ω_*c*_ = ω_*m*_ + *S*_1_(0). This is not the resonance condition for *H*_*S*_ in weak vibrational coupling theory. However, as we will soon show, if |*S*_1_(0)| ≪ 2Ω—a good definition of weak vibrational coupling—then ω_*c*_ = ω_*m*_ is a good approximation to resonance in the weak vibrational coupling regime.

In variational polaron theory, ω_*c*_ = ω_*m*_ + *S*_1_(0) is also not generally the resonance condition for . Consequently, “resonant” experiments should be modeled by a nonresonant Hamiltonian in the variational polaron frame. The resulting detuning between the cavity and molecular transition is Δ = ω_*m*_ – λ^*v*^ – ω_*c*_, and substituting in ω_*c*_ = ω_*m*_ + *S*_1_(0) yields
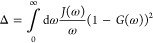
42Equation 42 shows that the system will be nonresonant when the vibrational and light–matter coupling strengths are strong and comparable because a large Δ *r*equires a simultaneously small *G*(ω) and large *J*(ω).

To complete the transformation, we must define *G̅* in eq 35. This is found through an optimization scheme detailed in Supporting Information Section S4, yielding
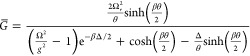
43and
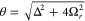
44is the polariton detuning in the nonresonant theory. Equation 44 shows that the resonance requires 2Ω_*r*_ ≫Δ. Since both  and Δ are functions of *G̅*, one must solve eqs 35, 38, 42, and 43 self-consistently.

The value of *G̅* in eq 43 and its dependence on the renormalized collective light–matter coupling Ω_*r*_ are essential to understanding how the rates and Lamb shifts scale with the number of molecules. The *N* scaling of *G̅* only depends on the size of Ω_*r*_ compared to the temperature
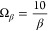
45There are slight variations in the *N* dependence of Δ and  if ω_0_ ≲ Ω_β_ or ω_0_ ≳ Ω_β_, but, these differences do not qualitatively change the *N* dependence of the master equation. Here in the main text, we present analysis for ω_0_ ≲ Ω_β_, but, as we show explicitly in Supporting Information Section S5, our main conclusions hold when ω_0_ ≳ Ω_β_ because the scaling of *G̅* with *N* is unchanged. For the typical molecular parameters introduced earlier, one requires temperatures below 7 K to enter the ω_0_ ≳ Ω_β_ regime, and so most molecular experiments are within ω_0_ ≲ Ω_β_.

**Figure 3:**
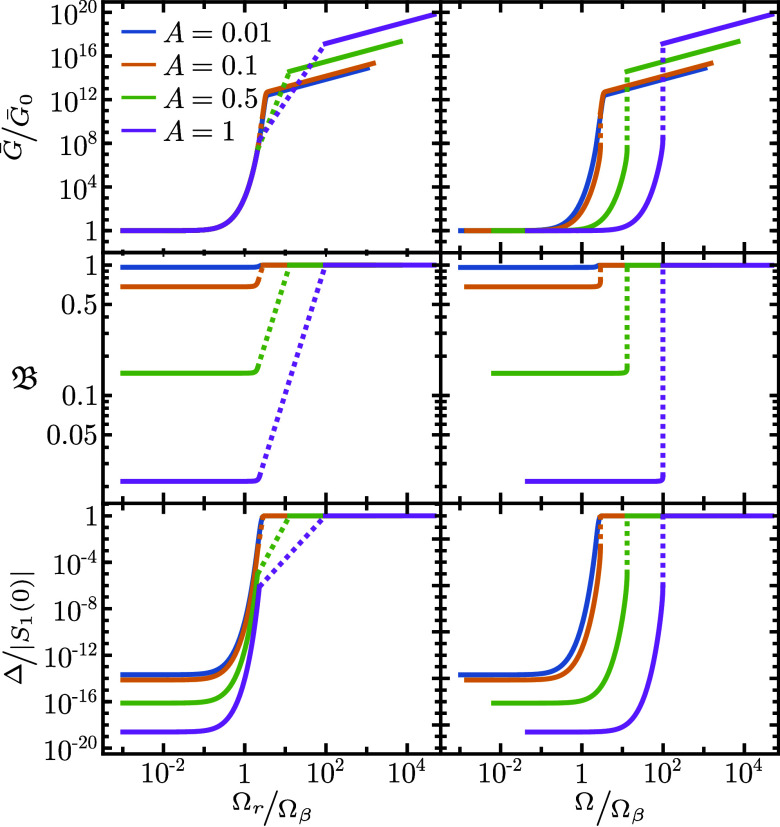
Variational parameter *G̅*, light–matter coupling renormalization , and detuning Δ are plotted against Ω_*r*_/Ω_β_ (left-hand column) and Ω/Ω_β_ (right-hand column) in the experimentally relevant regime with ω_0_ < Ω_β_. Data in the two columns are the same but plotted on different *x*-axes. Line color indicates vibrational coupling strength, *A*, with values given in the legend. The dotted lines are discontinuities. In the right-hand column, the discontinuities occur along the *y*-coordinates because for strong vibrational coupling (here *A* ≥ 0.1), the parameters are discontinuous near Ω_*r*_ = Ω_β_. In the left-hand column, the discontinuities are also along the *x*-coordinates because  is used in the definition of Ω_*r*_ = Ω. We have used *p* = 3 in the spectral density in eq 4. Other parameters: the typical molecular values discussed previously.

In Figure 3, we show *G̅*, , and Δ as a function of Ω_*r*_/Ω_β_. There are two distinct regimes, demarcated by Ω_*r*_ ∼ Ω_β_ and with a transitory region near the boundary. One can show that
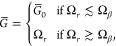
46where
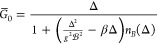
47Equation 47 is independent of *N* which occurs because of the dark state contribution (Ω^2^/*g*^2^ – 1)*e*^–βΔ/2^ in the denominator of eq 43. Without dark states, one would instead find that . As we will prove when we discuss dephasing, this fact explains why the decoherence rate is independent of *N* in Figure 1c for Ω_*r*_ ≲ Ω_β_.

Recent experiments^6,26^ have Ω_*r*_ ≤ 4.2 and 20.7 meV, and so for room-temperature experiments where Ω_β_ = 258 meV, the relevant regime is Ω_*r*_ ≲ Ω_β_. For typical molecular parameters, one finds that . Since the integrand of Δ in eq 42 scales with , Δ is negligible in this regime—as also shown in Figure 3—and so for typical molecular parameters, one can safely take the resonant limit of the variational polaron theory. The resonant value of  is
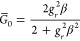
48Since  is much smaller than ω_0_ for typical molecular parameters, the WCME is expected to be very inaccurate for Ω_*r*_ ≲ Ω_β_. Moreover, in this regime, Figure 3 shows that the light–matter coupling can be heavily suppressed by  for strong vibrational coupling.

In the regime less relevant to recent experiments,^6,26^ Ω_*r*_ ≳ Ω_β_, we find  with the proportionality becoming an equality if the system is approximately resonant, 2Ω_*r*_ ≫ Δ. In this very strong light–matter coupling regime (or very low-temperature regime), *G̅* may be large enough that *G*(ω) ≈ 0 for all phonon frequencies ω that contribute to the dynamics. In this regime, the WCME will be accurate and  ≈ 1. However, also in this regime, Figure 3 shows that the detuning becomes equal to the vibrational reorganization energy. Consequently, one must assess whether the system is resonant by comparing the size of 2Ω_*r*_ to Δ = −*S*_1_(0).

In Table 1, we summarize the ω_0_ ≲ Ω_β_ regime of the variational transformation. Until the final section of this paper, we now enforce resonance, Δ ≡ 0, because this describes the most experimentally relevant parameter regimes and substantially simplifies the presentation of equations. In the final section, we summarize important corrections in the nonresonant regime.

**Table 1:** Variational Polaron Transformation for the Different Parameter Regimes^a^

ω_0_ ≲ Ω_β_		
Ω_*r*_ ≲ Ω_β_	Ω_*r*_ ≳ Ω_β_	
	2Ω_*r*_ ≲ |*S*_1_(0)|	2Ω_*r*_ ≫ |*S*_1_(0)|
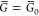	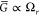	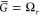
*G*(ω) ≈ 1	*G*(ω) ≈ 0	*G*(ω) ≈ 0
Δ = 0	Δ ≠ 0	Δ = 0

a The experimentally relevant regime has both ω_0_ ≲ Ω_β_ and Ω_*r*_ ≲ Ω_β_. *G*(ω) and Δ values shown are for the typical molecular parameters. For a similar breakdown in the regime ω_0_ ≳ Ω_β_, see Supporting Information Section S5.

## Variational Polaron Master Equation

We are now in a position to derive the Redfield master equation in the variational polaron frame. As a second-order perturbation in  given in eqs 39 and 40, we expect the VPME to have three distinct contributions. First, a displacement-type master equation , which by comparison of eq 39 to eq 3 will be identical to the WCME in eq 11 but with the replacement *J*(ω) → *J*(ω)(1 – *G*(ω))^2^ in the Fourier transforms of the correlation functions. Second, a polaron-type master equation , and finally, a variational-type contribution .

Rather than obfuscating the text by stating the general nonsecular VPME—which we give in Supporting Information Section S6—we will instead move onto discussing the transition rates, dephasing rates, and Lamb shifts predicted by the VPME. Analogously to the WCME in eq 16, the relevant part of the VPME is
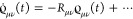
49where ϱ_μν_(*t*) = ⟨μ|ϱ_S_(*t*)|ν⟩ is an element of the variational frame density operator ϱ_S_(*t*) = Tr_B_[*U*ρ(*t*)*U*^†^] and
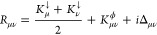
50where capitalized symbols are the equivalent quantities in the VPME to the corresponding lowercase symbols in eq 17 for the WCME. The loss rates can be written as summations of the transition rates
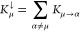
51and the Lamb-shifted transition frequencies are
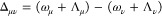
52

### Transition Rates

The displacement-type and variational-type master equations generate single-phonon processes, while the polaron-type master equation generates single- and multi-phonon processes. After collecting all terms, one finds that the transition rates are
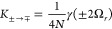
53
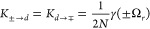
54and *K*_*d*→*d*≠*d*′_ = *k*_*d*→*d*≠*d*′_ is equal to the WCME (single-phonon) rate in eq 23. We have defined γ(ν) = 2Re[Γ(ν)] with
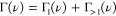
55where Γ_1_(ν) is the Fourier transform of the single-phonon bath correlation function, with real and imaginary parts given in eqs 14 and 15, respectively. The Fourier transform of the multi-phonon bath correlation function is
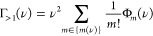
56with {*m*(±Ω_*r*_)} = {2, 3, 4, ...} and {*m*(±2Ω_*r*_)} = {3, 5, 7, ...}, and we have defined
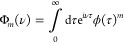
57as the Fourier transform of the *m*th power of the phonon propagator
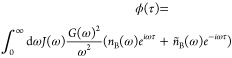
58

The *m*th term in the summation in eq 56 is the contribution of processes involving *m* phonons. The total loss rates from the eigenstates are
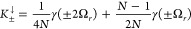
59
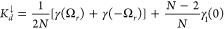
60

Compared with transition rates in the WCME in eqs 24 and 25, transition rates predicted by the VPME differ in two important ways. First, by the replacement Ω → Ω_*r*_ in the transition energies, the effects of which are well demonstrated in Figure 3. Second, by the introduction of multi-phonon transitions.

**Figure 4:**
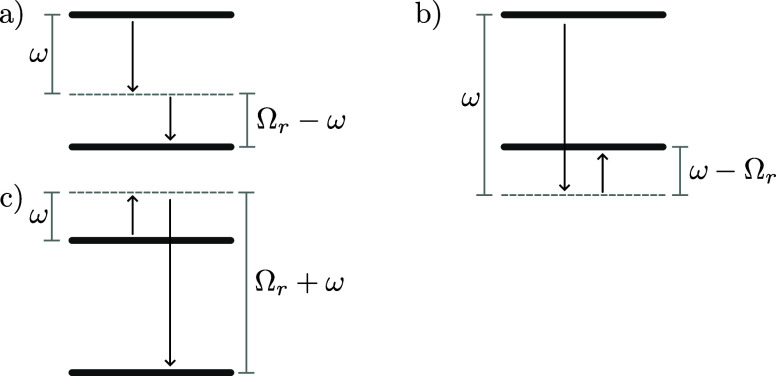
Transitions described by the integrands in eq 61. The downward and upward arrows denote emission and absorption of a phonon of the indicated energy, respectively. (a–c) Correspond to the first, second, and third terms of eq 61, respectively, and ω is integrated between the limits shown in eq 61. Since the transition energy is Ω_*r*_, the upper and lower states could be |+⟩ and |*d*⟩, or |*d*⟩ and |−⟩.

The effect of multi-phonon transitions depends on the size of Ω_*r*_ compared to the high-frequency cutoff ω_0_. To demonstrate why, it is helpful to expand the bath correlation function associated with two phonon decay into its contributions. For decay processes at transition frequency Ω_*r*_, the two-phonon contributions to the rates are proportional to
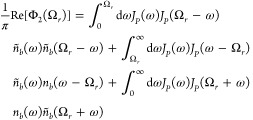
61where *J*_*P*_(ω) = *J*(ω)*G*(ω)^2^/ω^2^. In Figure 4, we illustrate the transitions described by eq 61.

When Ω_*r*_ ≫ ω_0_, the transition rates are significantly suppressed by the high-frequency cutoff of the vibrational baths. In this regime, multi-phonon processes dominate over single-phonon processes because the process of decaying by emitting two phonons of energy less than Ω_*r*_—shown in Figure 4a—is substantially more probable than emitting a single phonon of energy Ω_*r*_. This is because  when Ω_*r*_ ≫ ω_0_ for typical spectral densities. The same arguments apply for decays and excitations through higher order phonon processes; however, unless the vibrations are very strong (*A* ≫ 1), processes involving more than two or three phonons will not significantly contribute.

On the other hand, when Ω_*r*_ ≲ ω_0_, the cutoff frequency of the bath does not have as great an effect on the rates. Whether or not multi-phonon processes are dominant in this regime, and how they scale with *N*, depends on the form of the spectral density *J*(ω). In general, one must evaluate the rate functions in eqs 53 and (54) to determine the contribution of multi-phonon processes when Ω_*r*_ ≲ ω_0_.

**Figure 5:**
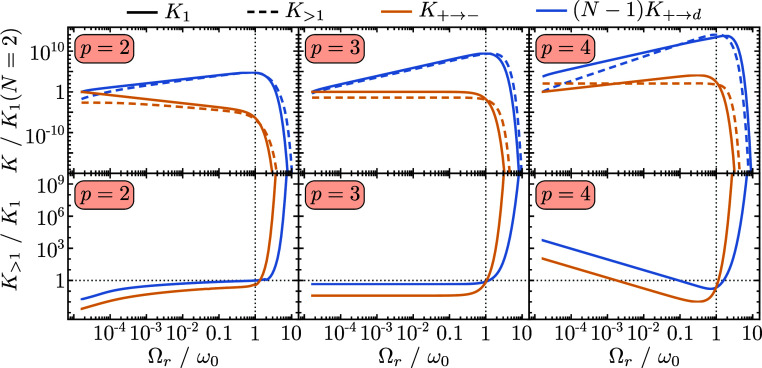
Transition rates from |+⟩ to |−⟩ (*K*_+→–_) and from |+⟩ to all dark states |*d*⟩ ((*N* – 1)*K*_+→*d*_) as a function of Ω_*r*_. In the top row, we show the single-phonon (*K*_1_—solid curves) and multi-phonon (*K*_>1_—dashed curves) contributions keeping up to third-order phonon interactions and normalized with respect to the single-phonon contribution when *N* = 2. In the bottom row, we plot the ratio of the single- and multi-phonon contributions shown in the panel above. Each column has a different Ohmicity, *p*, in the spectral density in eq 4. This figure shows that when Ω_*r*_ ≲ ω_0_, whether multi-phonon contributions dominate depends on the particular model, and that when Ω_*r*_ ≳ ω_0_ multi-phonon contributions always dominate. Parameters common to all panels: ω_0_ = 6 meV, *g* = 0.1 μeV, and *T* = 300 K. For Ohmicity *p* = 3 and *p* = 4, we use *A* = 0.083, while for *p* = 2, we use *A* = 0.0083, which give a similar value of  in each column.

In Figure 5, we calculate the multi-phonon contributions for Ohmicities *p* = 2, *p* = 3, and *p* = 4 (*p* is defined in eq 4) in the experimentally relevant regime Ω_*r*_ ≲ Ω_β_. One can see that each Ohmicity affords different ratios of single to multi-phonon contributions when Ω_*r*_ ≲ ω_0_, but in all cases the multi-phonon contributions strongly dominate when Ω_*r*_ ≫ ω_0_.

This discussion holds for all values of Ω_*r*_ compared to Ω_β_ so long as the system is resonant to a good approximation. In the regime less relevant to recent experiments,^6,26^ Ω_*r*_ ≳ Ω_β_, the *m* phonon contribution scales by a factor of *N*^–*m*/2^ differently to the same contribution when Ω_*r*_ ≲ Ω_β_ because when Ω_*r*_ ≳ Ω_β_,  as shown in eq 46. In the regime Ω_*r*_ ≳ ω_0_, the additional factor of *N*^–*m*/2^ will not change the fact that multi-phonon processes will be exponentially more probable than single-phonon processes.

This leads us to our first main result illustrated in Figure 1b. In the large *N* limit, the dark states become population sinks, and, if Ω_*r*_ ≳ ω_0_, the transitions are strongly dominated by multi-phonon processes.

### Dephasing Rates

The dephasing rates have contributions from the displacement-type and polaron-type master equations but not from the variational-type master equation. One finds that the overall dephasing rate of the coherence between states |μ⟩ and |ν⟩ is
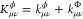
62where *k*_μν_^ϕ^ is the single-phonon contribution arising from the displacement-type interaction, exactly equal to the WCME dephasing rate in eq 26, and
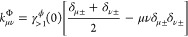
63is the polaron-type dephasing. The first term of *k*_μν_^Φ^ is the contribution from virtual self-transitions while the second term is from other pure dephasing processes. The function γ_>1_^ϕ^(0) = 2Re[Γ_>1_^ϕ^(0)] where
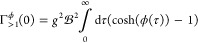
64describes multi-phonon dephasing processes. Using eq 26 for *k*_μν_^ϕ^ and (63) for *k*_μν_^Φ^, we find the following dephasing rates of the coherences
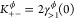
65
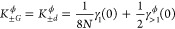
66
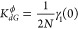
67
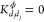
68

Notice that multi-phonon dephasing contributes to *K*_+–_^ϕ^—whereas for symmetry reasons single-phonon dephasing did not—and to *K*_±*G*_^ϕ^ which contributes to the polariton line widths.

By expanding cosh(ϕ(τ)) in eq 64 as a series in ϕ(τ), one finds that polaron-type dephasing is caused by processes with even numbers of phonons. The lowest order contribution is of second order
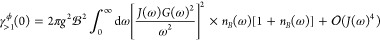
69which describes simultaneous phonon absorption and emission at all possible frequencies. This leading order contribution of the multi-phonon dephasing has the following scaling with *N*
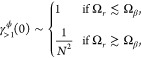
70which follows using eq 46 in eq 69.

In the variational polaron frame, the absorption spectrum of the cavity is described by eq 30 but using the quantities from the VPME. For instance, the line widths of the polariton peaks are equal to 2Re[*R*_±*G*_] = *K*_±_^↓^ + 2*K*_±*G*_^ϕ^. In the experimentally relevant regime, Ω_*r*_ ≲ Ω_β_, one finds from eqs 66 and 70 that the leading order contribution to the line widths is multi-phonon dephasing independent of *N*, which is a novel result of our theory.

On the other hand, when Ω_*r*_ ≳ Ω_β_, the leading order contribution is single-phonon dephasing scaling as γ_1_(0)/*N*. However, as discussed in the weak vibrational coupling section of this article, γ_1_(0) is zero or divergent for many spectral density choices. The next leading order term is two-phonon dephasing scaling as 1/*N*^2^.

Although less relevant to recent experiments,^6,26^ it is important to understand the leading order dephasing rate when Ω_*r*_ ≳ Ω_β_. To do so, we must clarify the zeros and divergences in the single-phonon contribution, γ_1_(0), given in eq 31. As we have discussed in the weak vibrational coupling section, these nonfinite results stem from an unjustified Markovian assumption: taking the infinite limit of the upper integration domain in the bath correlation function. Relaxing this assumption, one finds that the single-phonon pure dephasing rate is
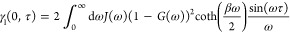
71

The Markovian limit is recovered by using the δ-function representation: lim_τ→∞_ sin(ωτ)/ω = πδ(ω).

Equation 71 should replace the factors of γ_1_(0) appearing in eqs 66 and 67. After a time *t*, the non-Markovian single-phonon rate γ_1_(0, τ) suppresses the coherences by a factor of
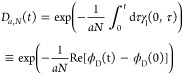
72compared to the initial value of the coherences. The factor of 1/(*aN*) in the exponent of eq 72, where *a* is constant, is the coefficient of γ_1_(0, τ) in either eq 66 or eq 67. For example, *D*_8,*N*_(*t*) is the suppression factor of ϱ_±*G*_(*t*) and ϱ_±*d*_(*t*) at time *t* due to γ_1_(0, τ)/(8*N*) appearing in eq 66. In 72 we have also defined the displacement-type phonon propagator
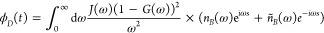
73which is analogous to the polaron-type phonon propagator in eq 58. The exponent of eq 72 is the so-called decoherence function^36^ which describes pure dephasing caused by emitting a finite frequency phonon at time 0 and reabsorbing the same phonon at time *t* ≥ 0. Note that *D*_1,1_(*t*) *exactly* describes the dephasing of the excited–ground state coherence of an isolated molecule with Hamiltonian *H*_mol_ in eq 41.^36^

**Figure 6:**
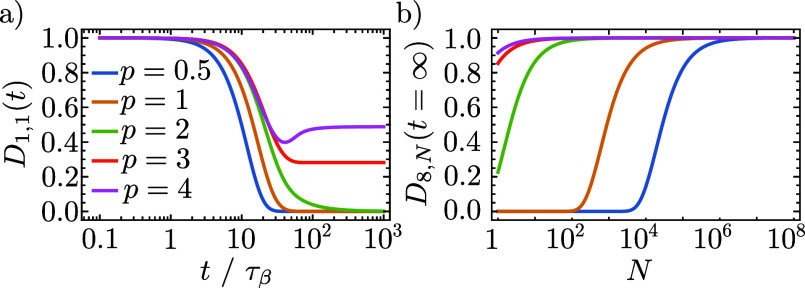
(a) *D*_1,1_(*t*) is shown as a function of time, and (b) *D*_8,*N*_(*t* = ∞) as a function of *N*. In both panels, the different colored curves correspond to different Ohmicity values *p* of the spectral density in eq 4, indicated in the legend, and τ_β_ = β/π is the thermal bath time.^36^ We enforce *G*(ω) = 0 for all parameters shown because we are interested only in the regime with Ω_*r*_ ≳ Ω_β_. Other parameters take the typical molecular values introduced earlier.

As shown in Figure 6a, *D*_1,1_(*t*) describes approximate exponential suppression of the coherences in time, but crucially, to a nonzero—albeit sometimes small—minimum value. This is an important distinction between Markovian and non-Markovian dephasing. On the one hand, Markovian dephasing—with a generic time-independent rate γ_M_—leads to a coherence suppression factor at time *t* of exp(−γ_M_*t*). On the other hand, non-Markovian dephasing—with a generic time-dependent rate γ_nM_(τ)—leads to a suppression factor of *exp*[−∫_0_^*t*^dτγ_nM_(τ)]. Therefore, while Markovian dephasing always suppresses the coherences to zero when *t* → ∞, the non-Markovian suppression factor may be greater than zero at *t* → ∞. This difference is particularly important for molecular experiments, where the regime in which γ_1_(0, τ) is relevant, Ω_*r*_ ≳ Ω_β_, can be reached only with *N* ≳ 10^12^ molecules. Figure 6b shows that the 1/*N* suppression within the exponent of *D*_*a*,*N*_(*t*) causes the long-time limit to approach unity, even for *N* ≪ 10^12^. Indeed, one finds that *D*_*a*,*N*_(*t*) → 1 at all times for relatively small values of *N*, such that single-phonon dephasing becomes negligible. This discussion suggests that all non-Markovian contributions to rates that scale inversely with *N*—which includes all rates in the VPME with the exception of γ_>1_^ϕ^(0) in the regime Ω_*r*_ ≲ Ω_β_—are negligible compared to the Markovian contributions.

This finding leads us to our second main result, as illustrated in Figure 1c. When Ω_*r*_ ≳ ω_0_, decoherence is dominated by dephasing, which, for typical molecular parameters, is a multi-phonon process involving all even orders of phonon interactions that scales independently of *N* when Ω_*r*_ ≲ Ω_β_, and a two phonon process scaling as 1/*N*^2^ when Ω_*r*_ ≳ Ω_β_.

### Lamb Shifts

We find the following expressions for the Lamb shifts
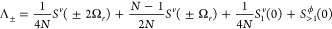
74
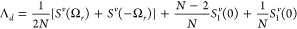
75and Λ_*G*_ = 0, where we have defined *S*^*v*^(ν) = *S*_1_^*v*^(ν) + *S*_>1_(ν) which has the single-phonon contribution
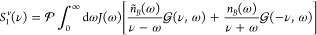
76with
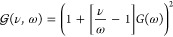
77and multi-phonon contribution *S*_>1_(ν) = Im[Γ_>1_(ν)] where Γ_>1_(ν) is given in eq 56.

The first two terms of eqs 74 and 75 arise from transitions between states, with the (*N* – 2)*S*_1_^*v*^(0)/*N* contribution in eq 75 arising from transitions between degenerate dark states. The third terms of eqs 74 and 75 arise from virtual transitions from a state back to itself, and the final term of eq 74 contains a contribution from virtual multi-phonon transitions, *S*_>1_^ϕ^(0) = Im[Γ_>1_^ϕ^(0)], where Γ_>1_^ϕ^(0) is given in eq 64.

In the large *N* limit, the Lamb shifts in eqs 74 and 75 are dominated by contributions from single-phonon transitions involving the large numbers of dark state, such that
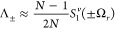
78
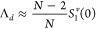
79

In the WCME, we found that the polariton shifts were negligible compared to the Rabi splitting because, in the WCME, when |ν|≫ ω_0_ the relevant function *S*_1_(ν) in eq 15 was inversely dependent on ν. Consequently, *S*_1_(±Ω) and *S*_1_(±2 Ω) vanished in the large *N* limit. However, in the variational polaron theory, the relevant function is instead *S*_1_^*v*^(ν) in eq 76, which generally has a different dependence on frequency owing to the factors of .

We are able to evaluate *S*_1_^*v*^(ν) when |ν|≫ ω_0_ in the limits Ω_*r*_ ≲ Ω_β_ and Ω_*r*_ ≳ Ω_β_ because, for typical molecular parameters, eq 46 indicates that we can approximate *G*(ω) = 1 and *G*(ω) = 0 in these limits, respectively. Within these parameter regimes, one finds that
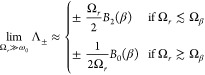
80where
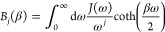
81

Equation 80 shows that in the parameter regime relevant to recent experiments,^6,26^ Ω_*r*_ ≲ Ω_β_, single-phonon Lamb shifts modify the polariton energies to
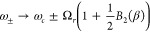
82for example, for the typical molecular parameters at room temperature, *B*_2_(β) = 0.634 when *p* = 3, which is a significant Lamb shift [for *p* < 2, *B*_2_(β) diverges but this is because we have approximated *G̅* = 0 to arrive at eq 80, while in fact it is nonzero but small].

Returning to the leading order contribution to the dark state Lamb shift in eq 79, using eq 76 one finds that in the large *N* limit
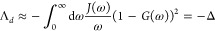
83which is equal to the negative of the detuning in . This Lamb shift is negligible for typical molecular parameters in the limit Ω_*r*_ ≲ Ω_β_ because 1 – *G*(ω) ∝ *G̅*, but may be large when Ω_*r*_ ≳ Ω_β_ if the system is nonresonant.

This discussion brings us to our third main result, as illustrated in Figure 1d. When Ω_*r*_ ≳ ω_0_ and for typical molecular parameters, if Ω_*r*_ ≲ Ω_β_ the polaritons are Lamb shifted by equal and opposite amounts proportional to Ω_*r*_, while, if Ω_*r*_ ≳ Ω_β_, the dark state is Lamb shifted by an amount equal to the vibrational reorganization energy.

## Non-resonance

We now briefly summarize the effects of nonresonance on our three main conclusions summarized in Figure 1 regarding multi-phonon transitions, dephasing, and Lamb shifts. We derive these results from the nonresonant VPME given in Supporting Information Section S7. Recall that inadvertent nonresonance occurs if both |*S*_1_(0)| ≳ 2Ω_*r*_ and Ω_*r*_ ≳ Ω_β_ are satisfied (assuming that ω_*c*_ = ω_*m*_ + λ) which requires strong vibrational coupling and either strong light–matter coupling or very low temperature. One could also avoid inadvertent nonresonance by building a wedge-shaped cavity and continuously tuning the mode energy until it becomes resonant with the matter system.^40^

Regarding multi-phonon transitions into the dark states, when the system is nonresonant, transitions between the polariton states and the dark states become
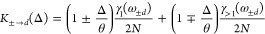
84where θ is the polariton detuning in eq 44, ω_±*d*_ = ± (θ ∓Δ)/2 are the transition energies, and γ_1_(ν) and γ_>1_(ν) are the rate functions from the resonant theory. Consequently, in far off-resonant systems where Δ is comparable or larger than 2Ω_*r*_, single-phonon transitions from the upper and lower polaritons to the dark states will be, respectively, enhanced and suppressed, while the opposite is true for multi-phonon transitions. This may be significant in the regime θ/2 ≫ ω_0_ when multi-phonon processes dominate, as discussed in Figure 5.

Regarding multi-phonon dephasing, the nonresonant expression is
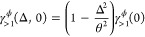
85where γ_>1_^ϕ^(0) is the resonant multi-phonon dephasing rate defined through eq 64. In far off-resonant systems, the dephasing rate will be slower than anticipated from the resonant theory.

Lastly, nonresonance does not affect Lamb shifts. This is because nonresonance requires Ω_*r*_ ≳ Ω_β_, and in this limit, the only non-negligible Lamb shift is to the dark state energy, originating from transitions between degenerate dark states. Since nonresonance does not affect properties of the dark states, this Lamb shift is also unaffected.

The nonresonant corrections in eqs 84 and 85 scale to leading order as . Since nonresonance requires Ω_*r*_ ≳ Ω_β_, and at room temperature Ω_β_ = 0.258 eV, nonresonant corrections will only appear for very strong vibrational coupling strengths, or in very low temperature experiments. For example, at room temperature and for an Ohmic spectral density with ω_0_ = 6 meV, one requires *A* > 30 for , which is orders of magnitude larger than the typical molecular value of *A* = 0.083. Conversely, for *A* = 0.083, one requires *T* < 0.8 K.

## Conclusions

By deriving the Redfield equation in the variational polaron frame, we have shown that multi-phonon processes and vibrational suppression of the light–matter coupling are important phenomena in molecular polaritonics.

Vibrational displacement interactions cause transitions between the upper polariton, lower polariton, and dark states. When the collective light–matter coupling is smaller than the high-frequency cutoff of the vibrations (Ω_*r*_ ≲ ω_0_), we have shown that whether multi-phonon processes dominate transition rates depends strongly on the spectral density. Conversely, when Ω_*r*_ ≫ ω_0_—a parameter regime now accessible to experiments^26^—one finds that multi-phonon processes always dominate. An important result found in refs (19 and 20), valid for this was that the dark states act as population sinks when there are a large number of molecules. This result holds at strong vibrational coupling, but we found here that the transfer is carried out through single *and* multi-phonon processes when Ω_*r*_ ≲ ω_0_ and almost exclusively by multi-phonon processes when Ω_*r*_ ≫ ω_0_.

Vibrational displacement interactions also cause the dephasing of eigenstate coherences. This is particularly important in the limit Ω_*r*_ ≫ ω_0_ where the contribution of the decay rates to decoherence is exponentially suppressed with increasing *N* such that decoherence is dominated by dephasing. We found that dephasing is always a multi-phonon process to lead to order in *N*. When Ω_*r*_ ≲ Ω_β_—the regime relevant to recent experiments^6,26^—one finds that dephasing is independent of *N*, while when Ω_*r*_ ≳ Ω_β_, dephasing scales as 1/*N*^2^. The *N*-independence of the dephasing rates when Ω_*r*_ ≲ Ω_β_ originates from the contribution of the *N* – 1 dark states to the free energy of the system, manifesting as *G̅* being independent of *N* in eq 46. This is an important and novel role for dark states in molecular polaritonics. This is a substantially different result than one finds in the weak coupling theory where dephasing is zero or divergent for all but Ohmic spectral densities.^19^

Regarding Lamb shifts, we showed that when Ω_*r*_ ≲ Ω_β_, the polariton energies can be significantly Lamb shifted even for only moderately strong vibrational coupling. This prediction cannot be obtained from the weak vibrational coupling theory and arises due to transitions from the polaritons into the dark states. We derived a simple expression for the Lamb shifted polariton energies, given in eq 82, valid for when Ω_*r*_ ≫ ω_0_. We also showed that if *N* is increased or the temperature is reduced such that Ω_*r*_ ≳ Ω_β_, then the polariton Lamb shifts become negligible but the dark states become Lamb shifted by an amount equal to the negative of the cavity–molecule detuning. To the best of our knowledge, this is the first calculation of vibrational Lamb shifts in this system.

Finally, we briefly discussed corrections to the multi-phonon transition and dephasing rates when the model is nonresonant. We found that nonresonant effects are likely to be negligible for molecular experiments unless they are performed at temperatures below 1 Kelvin.

In this paper, we have uncovered key effects of strong vibrational coupling in molecular polaritonics. There are many unsolved challenges for analytical modeling. For instance, intramolecular couplings can change which energy-transfer pathways are dominant,^24^ neglecting permanent dipole interactions has been shown to cause unphysical dips in polaritonic energy surfaces,^41^ and, in the ultrastrong light–matter coupling regime, the rotating wave approximation—responsible for removing the counter rotating light–matter interactions in eq 1—is erroneous.^42^ As well as mathematical challenges, counter rotating interactions introduce conceptual challenges because the number of excitations is no longer a good quantum number, and so distinctions between modeling the molecular excitation as bosonic (e.g.,^19,24^) or as a two-level system (e.g., presented here and in ref (21)) emerge.

## Data Availability

The data that support the findings of this study are openly available at see ref (43).

## Supporting Information Available

Derivations of some equations; total nonsecular master equations for the resonant and nonresonant models; and regime ω_0_ ≳ Ω_β_ (PDF)
