# Supplementary material for: Influence of Strong Molecular Vibrations on Decoherence of Molecular Polaritons
Source: ACS Photonics. 2024 Nov 15;11(12):5215–28. doi: 10.1021/acsphotonics.4c01446 (PMC11660232; doi:10.1021/acsphotonics.4c01446)
Supplement: Supplementary file 1 — ph4c01446_si_001.pdf [file ph4c01446_si_001.pdf]

Supporting Information: Influence of strong molecular vibrations on decoherence of molecular polaritons

Dominic M. Rouse,[1,*] Erik M. Gauger,[2] and Brendon W. Lovett[3,†]

[1]School of Physics and Astronomy, University of Glasgow, Glasgow, G12 8QQ UK
[2]SUPA, Institute of Photonics and Quantum Sciences, Heriot-Watt University, Edinburgh, EH14 4AS, UK
[3]SUPA, School of Physics and Astronomy, University of St Andrews, St Andrews, KY16 9SS, UK

SECTION 1. DATA FROM REF. [1] (REF. [6] IN THE MAIN TEXT).

In this appendix we summarize the necessary data from Ref. [1] to calculate Ω_r and Δ in the non-resonant variational polaron master equation.

From the 'Materials and methods' section of Ref. [1] one finds that the light–matter coupling is $g = 10.6$ neV, $N \in [0.21, 16] \times 10^{10}$ across the experiments which were performed at room temperature. The dephasing rate is $(1.68/N)$ meV which, due to the $1/N$ scaling, means that we must assume an Ohmic spectral density. Using Eq. (4) with $p = 1$ in Eq. (31) one finds that $A = (4\beta/\pi) \times 1.68$ meV $= 0.083$.

SECTION 2. CAVITY LEAKAGE AND NON-RADIATIVE MOLECULAR DECAY

In this appendix we derive the contribution of photon leakage from the cavity and non-radiative molecular decays to the transition and dephasing rates derived in the main text. These processes cause transitions from the single excitation manifold eigenstates $|+\rangle$, $|-\rangle$, and $\{|d\rangle\}$, to the global ground state $|G, n = 0\rangle$.

These processes can be added to the model using Lindblad dissipators, because the Rabi frequency is small compared to the molecular energy, $\Omega_r \ll \omega_m$ [2]. We add the following term to the right hand-side of the variational polaron master equation in Eq. (49),

$$\gamma_c \mathcal{L}_a[\varrho_S(t)] + \gamma_{\rm nr} \sum_{i=1}^{N} \mathcal{L}_{\sigma_i^-}[\varrho_S(t)], \qquad (S.1)$$

where γ_c is the photon leakage rate from the cavity, $\gamma_{\rm nr}$ is the non-radiative decay rate of the molecules, and

$$\mathcal{L}_O[\varrho_S(t)] = O\varrho_S(t)O^\dagger - \frac{1}{2}\{O^\dagger O, \varrho_S(t)\}, \qquad (S.2)$$

is a Lindblad superoperator.

One finds that the transition rates between the single excitation eigenstates and the global ground state are

$$K_{\pm\to G} = \frac{\gamma_c + \gamma_{\rm nr}}{2}, \qquad (S.3)$$

$$K_{d\to G} = \gamma_{\rm nr}. \qquad (S.4)$$

The loss rates of the eigenstates will also contribute to the decoherence rate of the coherence between the eigenstate and the global ground state, which in turn contributes to the width of the polariton peaks in the spectrum. The decoherence rate between the single excitation state $|\mu\rangle$ and global ground state $|G, 0\rangle$ (described by Eq. (50)) now has an additional contribution of $K_{\mu\to G}/2$.

SECTION 3. WIDTH OF POLARITON PEAKS IN ABSORPTION SPECTRUM

In this appendix we derive an expression for the widths of the polariton peaks in terms of quantities discussed in the main text.

The absorption spectrum of the cavity is [3, 4]

$$\mathcal{A}(\omega) = {\rm Re}\int_0^\infty {\rm d}\tau\ {\rm e}^{i\omega\tau} \lim_{t\to\infty} \langle \mathbf{E}_+(\mathbf{R}, t+\tau) \cdot \mathbf{E}_-(\mathbf{R}, t)\rangle, \qquad (S.5)$$

which is the Fourier transform of the correlation function between the positive and negative components of the electric field,

$$\mathbf{E}_+(\mathbf{R}, t) = +i\mathbf{e}\sqrt{\frac{\omega_c}{2V}}a(t){\rm e}^{i\omega_c R}, \qquad (S.6)$$

and $\mathbf{E}_-(\mathbf{R}, t) = \mathbf{E}_+(\mathbf{R}, t)^\dagger$, where $\mathbf{R}$ is the position of the detector, V and $\mathbf{e}$ are the quantization volume and polarization vector of the cavity mode, and $a(t)$ is the photon annihilation operator in the Heisenberg picture. Note that we used natural units with the speed of light $c = 1$.

Assuming that the detector is far enough from the dipole that we can ignore the phase factors, the absorption spectrum is

$$\mathcal{A}(\omega) = \mathcal{A}_0{\rm Re}\int_0^\infty {\rm d}\tau\ {\rm e}^{i\omega\tau} \lim_{t\to\infty} \langle a(t+\tau)a^\dagger(t)\rangle, \qquad (S.7)$$

where $\mathcal{A}_0 = \omega_c/(2V)$. Note that the area of the spectrum is a constant,

$$\int_0^\infty {\rm d}\omega\ \mathcal{A}(\omega) = \pi\mathcal{A}_0\left(1 + \langle a^\dagger(\infty)a(\infty)\rangle\right). \qquad (S.8)$$

* dominic.rouse@glasgow.ac.uk
† bwl4@st-andrews.ac.uk

Using the quantum regression theorem [5] in the variational polaron frame, one finds that

$$\lim_{t\to\infty}\langle a(t+\tau)a^\dagger(t)\rangle = \mathrm{Tr}\left[a\zeta(\tau)\right], \tag{S.9}$$

where

$$\zeta(\tau) = \lim_{t\to\infty}\mathrm{Tr}_B\left[U_0(\tau)a^\dagger\varrho(t)U_0^\dagger(\tau)\right], \tag{S.10}$$

and $U_0(\tau)=\exp[-i\mathcal{H}\tau]$. The operator $\zeta(\tau)$ evolves with respect to τ under the same master equation as $\varrho(\tau)$—the variational polaron frame master equation—but has the modified initial state,

$$\zeta(0) = a^\dagger\varrho(\infty). \tag{S.11}$$

Within the $n=1$ single exciton manifold the only nonzero term in the trace in Eq. (S.9) comes from $\langle G,0|\,a = \langle G,1|$ such that

$$\lim_{t\to\infty}\langle a(t+\tau)a^\dagger(t)\rangle = \langle G,1|\zeta(\tau)|G,0\rangle \tag{S.12}$$

$$= \frac{1}{\sqrt{2}}\left[\zeta_{+G}(\tau)+\zeta_{-G}(\tau)\right], \tag{S.13}$$

where subscript 'G' refers to the zero excitation state $|G,0\rangle$. The matrix elements $\zeta_{\pm G}(\tau)$ evolve in τ identically to the time evolution of the coherences between the polariton states $|\pm\rangle$ and the ground state $|G,0\rangle$. Therefore, the absorption spectrum is related to the variational polaron frame master equation by

$$\mathcal{A}(\omega) = \frac{\mathcal{A}_0}{\sqrt{2}}\mathrm{Re}\int_0^\infty \mathrm{d}\tau\ \mathrm{e}^{i\omega\tau}\left[\zeta_{+G}(\tau)+\zeta_{-G}(\tau)\right], \tag{S.14}$$

with $\zeta_{\pm G}(0) = \varrho_{GG}(\infty)/\sqrt{2}$ obtained from Eq. (S.11). Notice that Eq. (S.14) yields $\int_0^\infty \mathrm{d}\omega\ \mathcal{A}(\omega) = \pi\mathcal{A}_0\varrho_{GG}(\infty)$ for the area of the spectrum. When this is compared to Eq. (S.8), we find that $\rho_{GG}(\infty) = 1 + \langle a^\dagger(\infty)a(\infty)\rangle$, which—because a physical solution requires $0\le\rho_{GG}(\infty)\le 1$ and $\langle a^\dagger(\infty)a(\infty)\rangle \ge 0$—implies that $\rho_{GG}(\infty)=1$ and $\langle a^\dagger(\infty)a(\infty)\rangle = 0$. That is, our expression is correct so long as all photons eventually leak from the cavity.

We must now evaluate the integral in Eq. (S.14) using the variational polaron frame master equation. To obtain results that we can interpret in terms of parameters in the main text we secularize the variational polaron frame master equation. In a secular master equation, coherences evolve generally as $\dot{\varrho}_{\mu\nu}(\tau) = -R_{\mu\nu}\varrho_{\mu\nu}(\tau)$ with solution

$$\varrho_{\mu\nu}(\tau) = \varrho_{\mu\nu}(0)\mathrm{e}^{-R_{\mu\nu}\tau}, \tag{S.15}$$

where $\mu\neq\nu$ and $R_{\mu\nu}$ is given in Eq. (50) for the variational polaron master equation. We can neglect the other terms within the ellipses in Eq. (49), resulting in Eq. (S.15), because we are only concerned with coherences $\mu\neq\nu$ *and* we have secularized the master equation.

One can now substitute Eq. (S.15) into Eq. (S.14), replacing $\varrho_{\pm G}(\tau)$ with $\zeta_{\pm G}(\tau)$ and using the initial conditions $\zeta_{\pm G}(0) = 1/\sqrt{2}$. Performing the integration yields Eq. (30) but with the WCME quantities replaced with the VPME quantities: $r_{pG}\to R_{pG}$ and $\delta_{pG}\to\Delta_{pG}$. The spectrum describes two Lorentzian distributions with maxima at the Lamb shifted polariton energies $\Delta_{\pm G} = \omega_\pm + \Lambda_\pm$, and full width half maxima equal to $2\mathrm{Re}[R_{\pm G}] = K_\pm^\downarrow + 2K_\pm^\phi$.

We note that Eq. (30) for the spectrum is only very approximately true in the variational polaron frame. For $\mathfrak{B}\ll 1$ we would expect the polariton peaks to have phonon sidebands. Moreover, Ref. [6] has recently shown that previous calculations of spectra in polaron frames are erroneous, because they miss contributions from the so-called irrelevant terms of the master equation in the Nakajima-Zwanzig formulation [7]. In any case, our calculation of the spectrum is only to illustrate the importance of the dephasing rates in the large N limit, and not to make an accurate calculation of the spectrum when $\mathfrak{B}\ll 1$.

SECTION 4. VARIATIONAL OPTIMISATION

In this section we derive the optimisation scheme that determines which variational parameters $\{\eta_{\mathbf{k}}\}$ give the unperturbed Hamiltonian $\mathcal{H}_S$ in Eq. (36) that most closely resembles the equilibrium state of the full Hamiltonian $\mathcal{H}$.

The equilibrium density operator of the model is $\exp(-\beta\mathcal{H})/Z$ where $Z=\mathrm{Tr}_B[\exp(-\beta\mathcal{H})]$ is the partition function, which has a free energy $F=-\beta^{-1}\ln Z$ that is minimised in equilibrium. Substituting the partition function into F and then using the Feynman-Bogoliubov-Peierls upper bound identity [8] we find that,

$$F = -\beta^{-1}\ln\mathrm{Tr}_B\left[\mathrm{e}^{-\beta(\mathcal{H}_0+\mathcal{H}_{SB})}\right] \le -\beta^{-1}\ln\mathrm{Tr}_B\left[\mathrm{e}^{-\beta\mathcal{H}_0}\right] + \frac{\mathrm{Tr}_B[\mathcal{H}_{SB}\mathrm{e}^{-\mathcal{H}_0}]}{\mathrm{Tr}_B[\mathrm{e}^{-\mathcal{H}_0}]} + \mathcal{O}\left(\mathcal{H}_{SB}^2\right), \tag{S.16}$$

where $\mathcal{H}_0 = \mathcal{H}_S + \mathcal{H}_B$ is the unperturbed Hamiltonian. By construction, $\mathrm{Tr}_B[\mathcal{H}_{SB}\exp(-\beta\mathcal{H}_0)] = 0$, and further ignoring terms of second order and greater in the perturbation leads to

$$F \lesssim -\beta^{-1}\ln\mathrm{Tr}_B\left[\mathrm{e}^{-\beta\mathcal{H}_0}\right] \equiv F_{\mathrm{FBP}}. \tag{S.17}$$

Eq. (S.17) indicates that the free energy of system plus bath is less than or equal to the free energy of the unperturbed variational polaron frame Hamiltonian, F_{FBP}. By choosing $\{\eta_{\mathbf{k}}\}$ to minimise F_{FBP}, $\mathcal{H}_0$ will be defined such that it gives the closest representation of the equilibrium state as permitted by a polaron type transformation. Therefore, the equilibrium contribution of $\mathcal{H}_{SB}$ to the dynamics will be minimal, and a theory perturbing in $\mathcal{H}_{SB}$ as accurate as possible through optimising $\{\eta_{\mathbf{k}}\}$.

Using the eigensystem of $\mathcal{H}_0$ we find

$$F_{\rm FBP} = -\frac{1}{\beta}\ln\left(1+\sum_{n=1}^{\infty}\left[2\cosh\left[\frac{\beta\theta_n}{2}\right]\mathrm{e}^{-\beta\left(\frac{\Delta}{2}+n\nu\right)} + (N-1)\,\mathrm{e}^{-\beta(\Delta+n\nu)}\right]\right)\mathrm{tr}\left[\mathrm{e}^{-\beta\mathcal{H}_B}\right], \quad \text{(S.18)}$$

where $\Delta = \omega_m - \lambda - \omega_c$ is the detuning and $\theta_n = \sqrt{\Delta^2 + 4\Omega_r^2 n}$. Throughout the main text we assume that there is one photon in the cavity which means that only the eigenstates with $n = 1$ contribute to the dynamics. The equivalent assumption here is to only take the $n = 1$ term in the summation in Eq. (S.18). We then minimise $F_{\rm FBP}$ with respect to $\eta_{\mathbf{k}}$ to find the optimal expression for $G(\omega_{\mathbf{k}}) = \eta_{\mathbf{k}}/f_{\mathbf{k}}$ given by Eqs. (35) and (43).

SECTION 5. VARIATIONAL OPTIMIZATION IN THE LOW TEMPERATURE REGIME

In the Main Text we focused on the experimentally relevant, room temperature regime with $\omega_0 \lesssim \Omega_\beta$. In this appendix we discuss differences when the temperature is low enough that $\omega_0 \gtrsim \Omega_\beta$. For a typical molecular high frequency cutoff of $\omega_0 = 6$ meV [9] this requires temperatures below 7 K.

Fig. S1 is similar to Fig. 3 in the Main Text but now within the low temperature regime with $\omega_0 \gtrsim \Omega_\beta$. There are two differences between the high and low temperature parameter regimes. (1) At low temperature, Fig. S1 demonstrates that the effects of increasing vibrational coupling strength are diminished because the baths are essentially 'frozen out'. (2) At low temperature, the size of Ω_r compared to both Ω_β and ω_0 are important for $\mathfrak{B}$ and Δ, whereas at high temperature only Ω_β was important. These differences do not change any of the qualitative conclusions we have drawn in the main text regarding the N scaling of the master equation. This is because in both Fig. 3 and Fig. S1, $\bar{G}$ is described by Eq. (46), and the system is always non-resonant to a good approximation when $\Omega_r \ll \Omega_\beta$.

SECTION 6. MASTER EQUATION DERIVATION

In this appendix we derive the non-secular Redfield equation in the variational polaron frame. The Redfield equation in the Schrödinger picture is

$$\partial_t\varrho(t) = -i\left[\mathcal{H}_S, \varrho(t)\right] - U_S(t)\int_0^\infty \mathrm{d}\tau\, \mathrm{tr}_B\left[\tilde{\mathcal{H}}_{SB}(t), \left[\tilde{\mathcal{H}}_{SB}(t-\tau), \varrho(t)\right]\right]U_S(t)^\dagger \quad \text{(S.19)}$$

where a tilde denotes operators transformed into the interaction picture, $\mathrm{tr}_B[\cdot]$ is a trace over the Hilbert spaces of the baths, and $U_S(t) = \exp(-i\mathcal{H}_S t)$. $\mathcal{H}_S$ is the system Hamilton in Eq. (36) and $\mathcal{H}_{SB} = \mathcal{H}_D + \mathcal{H}_P$ is the system–bath interaction given in Eqs. (39)–(40). For later algebraic ease we decompose the master equation into contributions from each interaction type,

$$\partial_t\varrho(t) = -i\left[\mathcal{H}_S, \varrho(t)\right] + \sum_{a,b\in\{D,P\}} L_{ab}[\varrho(t)], \quad \text{(S.20)}$$

where

$$L_{ab}\left[\varrho(t)\right] = - U_S(t)\int_0^\infty \mathrm{d}\tau\, \mathrm{tr}_B\left[\tilde{\mathcal{H}}_a(t), \left[\tilde{\mathcal{H}}_b(t-\tau), \varrho(t)\right]\right]U_S(t)^\dagger. \quad \text{(S.21)}$$

The superoperators $L_{DD}[\varrho(t)]$ and $L_{PP}[\varrho(t)]$ are the displacement–type and polaron–type master equations arising solely from $\mathcal{H}_D$ and $\mathcal{H}_P$, respectively, and $L_{PD}[\varrho(t)] + L_{DP}[\varrho(t)]$ is a superoperator unique to the variational polaron master equation.

We will now derive each master equation contribution in turn. We use subscripts $\{\alpha, \beta, \gamma, \delta\}$ to denote any eigenstate $|+\rangle$, $|-\rangle$ and $\{|d\rangle\}$ for $d \in \{d_1, \ldots, d_{N-1}\}$ whilst we use $\{p, q, r, s\}$ to label only the polaritons $|+\rangle$ and $|-\rangle$. Finally, we define the transition energies and eigenstate transition operators,

$$\omega_{\alpha\beta} = \omega_\alpha - \omega_\beta, \quad \text{(S.22)}$$

$$\Pi_{\alpha\beta} = |\alpha\rangle\langle\beta|. \quad \text{(S.23)}$$

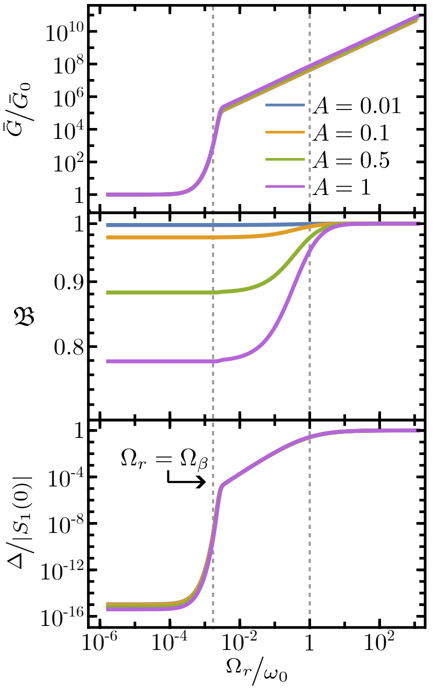

FIG. S1. Similar to Fig. (3) but here in the low temperature regime with $\omega_0 \gtrsim \Omega_\beta$. Parameters used: $g = 0.1$ μeV, $\omega_0 = 50$ meV, $T = 0.1$ K, and $p = 3$. Note that we use exaggerated values for ω_0 and T to clearly demonstrate the regime $\Omega_\beta < \Omega_r < \omega_0$.

A. Displacement–type contribution

Substituting $\mathcal{H}_D$ in Eq. (39) into $L_{DD}[\varrho(t)]$ yields the displacement–type master equation which has the same form as the second line of the weak vibrational coupling master equation in Eq. (11), except that the spectral density in the correlation functions is replaced with the displacement–type spectral density,

$$J_D(\omega) = J(\omega)\left(1 - G(\omega)\right)^2. \tag{S.24}$$

The origin of this difference can be seen by comparing the form of the couplings in the lab and variational frame displacement–type interactions in Eq. (3) and (39), respectively. That is, one makes the substitution $f_{\mathbf{k}} \to f_{\mathbf{k}} - \eta_{\mathbf{k}} = f_{\mathbf{k}}(1 - G(\omega_{\mathbf{k}}))$ to move from the weak coupling H_{SB} to the variational frame displacement–type interaction $\mathcal{H}_D$.

We find that

$$L_{DD}[\varrho(t)] = \sum_{\alpha,\beta,\gamma,\delta} c_{\alpha\beta\gamma\delta}\Gamma_1^D(\omega_{\delta\gamma})\left[\Pi_{\gamma\delta}\varrho_S(t), \Pi_{\alpha\beta}\right] + \text{H.c.}, \tag{S.25}$$

where $c_{\alpha\beta\gamma\delta}$ is defined in Eq. (12) and

$$\Gamma_1^D(\nu) = M_+\left[J_D(\nu)\right], \tag{S.26}$$

where

$$\mathrm{Re}\left(M_\pm\left[F(\nu)\right]\right) = \pi \times \begin{cases} \pm F(\nu)\tilde{n}_B(\nu) & \text{if } \nu \geq 0, \\ F(-\nu)n_B(-\nu) & \text{if } \omega < 0, \end{cases} \tag{S.27}$$

and

$$\mathrm{Im}\left(M_\pm\left[F(\nu)\right]\right) = \mathcal{P}\int_0^\infty \mathrm{d}\omega\ F(\omega)\left[\pm\frac{\tilde{n}_B(\omega)}{\nu - \omega} + \frac{n_B(\omega)}{\nu + \omega}\right], \tag{S.28}$$

for any function $F(\nu)$. The functional with a negative subscript, $M_-[F(\nu)]$, will be used in the variational–type master equation. In this notation, the Fourier transform of the single phonon correlation function given in Eq. (13) is $\Gamma_1(\nu) = M_+[J(\nu)]$.

B. Polaron–type contribution

Substituting $\mathcal{H}_P$ in Eq. (40) into $L_{PP}[\varrho(t)]$ yields,

$$L_{PP}\left[\varrho(t)\right] = \tag{S.29}$$

$$-\sum_{\alpha\beta pq}\Big(c_{\alpha\beta}^{P(-)}\Gamma^{P(-)}(\omega_{q\beta})\left[\Pi_{\alpha p}, \Pi_{\beta q}\varrho_S(t)\right] \tag{S.30}$$

$$+ c_{\alpha\beta}^{P(-)*}\Gamma^{P(-)}(\omega_{\beta q})\left[\Pi_{p\alpha}, \Pi_{q\beta}\varrho_S(t)\right] \tag{S.31}$$

$$+ c_{\alpha\beta}^{P(+)}\Gamma^{P(+)}(\omega_{\beta q})\left[\Pi_{\alpha p}, \Pi_{q\beta}\varrho_S(t)\right] \tag{S.32}$$

$$+ c_{\alpha\beta}^{P(+)*}\Gamma^{P(+)}(\omega_{q\beta})\left[\Pi_{p\alpha}, \Pi_{\beta q}\varrho_S(t)\right]\Big) + \text{H.c.}, \tag{S.33}$$

where the rate functions are

$$\Gamma^{P(\pm)}(\nu) = \frac{\Omega_r^2}{2N}\int_0^\infty \mathrm{d}\tau\ \mathrm{e}^{i\nu\tau}\left(\mathrm{e}^{\pm\phi(\tau)} - 1\right), \tag{S.34}$$

and the coefficients are,

$$c_{\alpha\beta}^{P(-)} = \sum_{i=1}^N u_{i\alpha}u_{i\beta}, \tag{S.35}$$

$$c_{\alpha\beta}^{P(+)} = \sum_{i=1}^N u_{i\alpha}u_{i\beta}^*. \tag{S.36}$$

The polaron–type phonon propagator $\phi(\tau)$ is defined in Eq. (58) and depends on the polaron–type spectral density function,

$$J_P(\omega) = J(\omega)\frac{G(\omega)^2}{\omega^2}. \tag{S.37}$$

By expanding $\exp(\phi(\tau)) \approx 1 + \phi(\tau)$ in Eq. (S.34), one can identify the single and multi phonon contributions of the polaron–type master, by using

$$\int_0^\infty \mathrm{d}\tau\ \mathrm{e}^{i\nu\tau}\left(\mathrm{e}^{\pm\phi(\tau)} - 1\right) = \pm M_+\left[J_P(\nu)\right] + \int_0^\infty \mathrm{d}\tau\ \mathrm{e}^{i\nu\tau}\left(\mathrm{e}^{\pm\phi(\tau)} - 1 \mp \phi(\tau)\right). \tag{S.38}$$

C. Variational–type contribution

Substituting $\mathcal{H}_D$ and $\mathcal{H}_P$ in Eqs. (39) and (40) into $L_{DP}[\varrho(t)] + L_{PD}[\varrho(t)]$ yields

$$L_{DP}[\varrho(t)] + L_{PD}[\varrho(t)] = \tag{S.39}$$

$$-\sum_{\alpha\beta\gamma p}\Big(c_{1,\alpha\beta\gamma}^V\Gamma^V(\omega_{\gamma p})\left[\Pi_{\alpha\beta}, \Pi_{p\gamma}\varrho_S(t)\right] \tag{S.40}$$

$$+ c_{2,\alpha\beta\gamma}^V\Gamma^V(\omega_{\beta\alpha})\left[\Pi_{\gamma p}, \Pi_{\alpha\beta}\varrho_S(t)\right] \tag{S.41}$$

$$+ c_{1,\alpha\beta\gamma}^V\Gamma^V(\omega_{\beta\alpha})\left[\Pi_{\alpha\beta}\varrho_S(t), \Pi_{p\gamma}\right] \tag{S.42}$$

$$+ c_{2,\alpha\beta\gamma}^V\Gamma^V(\omega_{p\gamma})\left[\Pi_{\gamma p}\varrho_S(t), \Pi_{\alpha\beta}\right]\Big) + \text{H.c.}, \tag{S.43}$$

where the rate function is

$$\Gamma_{\alpha\beta\gamma}^V(\nu) = \frac{\Omega_r}{\sqrt{2N}}M_-\left[J_V(\nu)\right], \tag{S.44}$$

the coefficients are,

$$c_{1,\alpha\beta\gamma}^V = \sum_{i=1}^N u_{i\alpha}u_{i\beta}^*u_{i\gamma}^* \tag{S.45}$$

$$c_{2,\alpha\beta\gamma}^V = \sum_{i=1}^N u_{i\alpha}u_{i\beta}^*u_{i\gamma}, \tag{S.46}$$

and the real and imaginary parts of $M_-[\cdot]$ are given in Eqs. (S.27)–(S.28). The variational–type spectral density function is

$$J_V(\omega) = J(\omega)\,(1 - G(\omega))\,\frac{G(\omega)}{\omega} \tag{S.47}$$

$$= \sqrt{J_D(\omega) J_P(\omega)}. \tag{S.48}$$

SECTION 7. NON RESONANCE

As shown in Fig. 3 in the Main Text, if $\Omega_r \gg \Omega_\beta$ then the detuning becomes equal to the vibrational reorganization energy, $\Delta = -S_1(0)$. If the vibrational coupling is strong enough that $|\Delta|$ is comparable to $2\Omega_r$, then the system must be described by a non-resonant Hamiltonian in the variational polaron frame.

If one cannot make the resonant approximation, then within the single photon and exciton manifold, $\mathcal{H}_S$ in Eq. (36) has the following polariton eigenstates,

$$|\pm\rangle = \mp\sqrt{N} U_\mp \,|G,1\rangle \pm U_\pm \,|B\rangle\,, \tag{S.49}$$

where

$$U_\pm = \frac{\pm 1}{\sqrt{2N}}\left(1 \pm \frac{\Delta}{\theta}\right)^{\frac{1}{2}}, \tag{S.50}$$

and

$$\theta = \sqrt{4\Omega_r^2 + \Delta^2}. \tag{S.51}$$

The polariton states have energies

$$\omega_\pm = \frac{\omega_m + \omega_c \pm \theta}{2}. \tag{S.52}$$

The $N-1$ degenerate dark states are described by the same vectors as in the resonant model and have an energy ω_m. This means that the transition energies in the non-resonant model are asymmetric, $\omega_+ - \omega_d = (\theta - \Delta)/2$ and $\omega_d - \omega_- = (\theta + \Delta)/2$.

We will now discuss the transition rates, dephasing rates, and Lamb shifts for the non-resonant variational polaron master equation. As in the resonant case in the main text, we derive these quantities by deriving the following element of the master equation

$$\dot{\varrho}_{\mu\nu}(t) = -R_{\mu\nu}(\Delta)\varrho_{\mu\nu}(t) + \dots, \tag{S.53}$$

where

$$R_{\mu\nu}(\Delta) = \frac{K_\mu^\downarrow(\Delta) + K_\nu^\downarrow(\Delta)}{2} + K_{\mu\nu}^\phi(\Delta) + i\Delta_{\mu\nu}(\Delta). \tag{S.54}$$

$R_{\mu\nu}(0) = R_{\mu\nu}$ is the resonant value given in Eq. (50). The loss rates can be written as summations of the transition rates,

$$K_\mu^\downarrow(\Delta) = \sum_{\alpha\neq\mu} K_{\mu\to\alpha}(\Delta), \tag{S.55}$$

and the Lamb shifted transition frequencies are

$$\Delta_{\mu\nu}(\Delta) = [\omega_\mu(\Delta) + \Lambda_\mu(\Delta)] - [\omega_\nu(\Delta) + \Lambda_\nu(\Delta)]\,, \tag{S.56}$$

where $\omega_\mu(\Delta)$ are the non-resonant eigenenergies.

We can anticipate the effects of detuning by considering the coupling operators in $\mathcal{H}_{SB} = \mathcal{H}_D + \mathcal{H}_P$ in Eqs. (39) and (40). In the large detuning limit, the lower polariton localizes onto the single photon state $|-\rangle \to |G,1\rangle$, whilst the upper polariton localizes onto the bright state $|+\rangle \to |B\rangle$. Therefore, in $\mathcal{H}_D$—which describes single phonon processes—the molecular coupling operator $\sigma_i^+\sigma_i^- = |e_i,0\rangle\langle e_i,0|$ only connects the dark states and upper polariton together, whilst in $\mathcal{H}_P$—which describes single and multi phonon processes—the coupling operator $a\sigma_i^+ = |e_i,0\rangle\langle G,1|$ only connects the dark states and upper polariton to the lower polariton. Consequently, in the large detuning limit, we expect single phonon processes involving the lower polariton to be suppressed, and multi phonon processes between the upper polariton and dark states to be suppressed. Additionally, since $a\sigma_i^+$ does not contain a state projector in the large detuning limit, we also expect multi phonon dephasing processes to be suppressed. As discussed in the main text, the leading order contribution to dephasing is multi phonon, and so non-resonance will lead to narrower polariton line widths than expected from the resonant theory.

A. Master equations

The master equations for the non-resonant model are modified slightly from those given in Appendix SECTION 6 for the resonant model, due to the modified eigenstates.

The displacement–type master equation has the same form as Eq. (S.25) with $c_{\alpha\beta\gamma\delta}$ given by Eq. (12). However, the $u_{i\alpha}$ now take the forms

$$u_{i\alpha} = \begin{cases} U_\pm & \text{if } \alpha = \pm, \\ u_{id} & \text{if } \alpha = d, \end{cases} \tag{S.57}$$

where $U_\pm$ are given in Eq. (S.50) and u_{id} are the same as in the resonant theory.

The non-resonant polaron–type master equation can be obtained from Eq. (S.29) with the replacements,

$$c_{\alpha\beta}^{P(\pm)} \to c_{\alpha\beta pq}^{P(\pm)} = 2N c_{\alpha\beta}^{P(\pm)} |U_{-p}||U_{-q}|, \tag{S.58}$$

where $c_{\alpha\beta}^{P(\pm)}$ are given in Eqs. (S.35)–(S.36).

Lastly, the non-resonant variational–type master equation can be obtained from Eq. (S.39) with the replacements,

$$c_{j,\alpha\beta\gamma}^V \to c_{j,\alpha\beta\gamma p}^V = \sqrt{2N} c_{j,\alpha\beta\gamma}^V |U_{-p}|, \tag{S.59}$$

for $j \in \{1,2\}$ where $c_{j,\alpha\beta\gamma}^V$ are given in Eqs. (S.45)–(S.46).

B. Transition rates

We will write the rates in terms of a generalized rate function,

$$\gamma_\Delta(\nu,\{a,b,c\}) = a\ \gamma_1(\nu) + b\ \gamma_{>1}^{\mathrm{even}}(\nu) + c\ \gamma_{>1}^{\mathrm{odd}}(\nu) \tag{S.60}$$

where a, b, and c are free parameters that may depend on Δ. $\gamma_1(\nu)$ describes single phonon processes and is given in Eq. (14). The remaining functions $\gamma_{>1}^{\mathrm{even}}(\nu) = 2\mathrm{Re}[\Gamma_{>1}^{\mathrm{even}}(\nu)]$ and $\gamma_{>1}^{\mathrm{odd}}(\nu) = 2\mathrm{Re}[\Gamma_{>1}^{\mathrm{odd}}(\nu)]$ describe even- and odd-ordered multi phonon processes, given generally by

$$\Gamma_{>1}^{M}(\nu) = \Omega_r^2 \sum_{\substack{m=2\\ m\in M}} \frac{1}{m!}\int_0^\infty \mathrm{d}\tau\ \mathrm{e}^{i\nu\tau}\phi(\tau)^m, \tag{S.61}$$

where $M \in \{\mathrm{even},\ \mathrm{odd}\}$ denotes only even or odd values of m are included in the summation, and $m = 2$ is excluded if $M \in$ odd. We also define the dimensionless parameter,

$$\varepsilon = \frac{\Delta}{\theta}, \tag{S.62}$$

which quantifies the detuning.

We find the non-resonant polariton-to-polariton transition rates,

$$K_{\pm\to\mp}(\Delta) = \frac{1}{4N}\gamma_\Delta\left(\pm\theta, \left\{1-\varepsilon^2, 2\varepsilon^2, 2\right\}\right), \tag{S.63}$$

which indicates that in the limit of large detuning, single phonon transitions between polaritons are suppressed, whilst even ordered multi phonon transitions are enhanced. The transition rates from the polaritons to the dark states are

$$K_{\pm\to d}(\Delta) = \frac{1}{2N}\gamma_\Delta\left(\pm\frac{\theta\mp\Delta}{2}, \{1\pm\varepsilon, 1\mp\varepsilon, 1\mp\varepsilon\}\right), \tag{S.64}$$

and transitions from dark states to the polaritons are the same up to a sign change on the first argument of the function on the right-hand-side which turns absorption processes into emission and vice-versa. The transition rates between degenerate dark states are the same as in the resonant model because dark states do not change off-resonance, $K_{d\to d'\neq d}(\Delta) = \gamma_1(0)/N$.

Eqs. (S.63)–(S.64) show that as the detuning increases, transitions between the upper polariton and the dark states are increasingly dominated by single phonon processes, whilst multi phonon processes become increasingly dominant for transitions between the lower polariton and the dark states. In the limit $\Omega_r \gg \omega_0$ this may have important implications because multi phonon processes are exponentially faster than single phonon processes. The total loss rates from the eigenstates are,

$$K_\pm^\downarrow(\Delta) = K_{\pm\to\mp}(\Delta) + (N-1)K_{\pm\to d}(\Delta), \tag{S.65}$$

$$K_d^\downarrow(\Delta) = K_{d\to+}(\Delta) + K_{d\to-}(\Delta) + \frac{N-2}{N}\gamma_1(0). \tag{S.66}$$

C. Dephasing rates

As in the resonant master equation, the dephasing rates have contributions from the displacement–type and polaron–type master equations:

$$K_{\mu\nu}^\phi(\Delta) = k_{\mu\nu}^\phi(\Delta) + k_{\mu\nu}^\Phi(\Delta). \tag{S.67}$$

The displacement–type contribution has the same form as in Eq. (26) but with the $c_{\alpha\beta\gamma\delta}$ coefficient in Eq. (12) now dependent on the non-resonant eigenbasis as described by Eq. (S.57). The polaron–type contribution—with $k_{\mu\nu}^\Phi(0)$ given for the resonant model in Eq. (63)—gains an overall prefactor dependent on the detuning,

$$k_{\mu\nu}^\Phi(\Delta) = \left(1-\varepsilon^2\right) k_{\mu\nu}^\Phi(0). \tag{S.68}$$

In terms of the generalized dephasing function,

$$\gamma_\Delta^\phi\left(\{a,b\}\right) = a\ \gamma_1(0) + b\ \gamma_{>1}^\phi(0), \tag{S.69}$$

where $\gamma_{>1}^\phi(0)$ is the multi phonon dephasing rate function defined through Eq. (64), the dephasing rates are

$$K_{+-}^\phi(\Delta) = \gamma_\Delta^\phi\left(\left\{\frac{1}{2N}\varepsilon^2, 2\left(1-\varepsilon^2\right)\right\}\right), \tag{S.70}$$

$$K_{\pm G}^\phi(\Delta) = \gamma_\Delta^\phi\left(\left\{\frac{1}{8N}\left(1\pm\varepsilon\right)^2, \frac{1}{2}\left(1-\varepsilon^2\right)\right\}\right), \tag{S.71}$$

$$K_{\pm d}^\phi(\Delta) = \gamma_\Delta^\phi\left(\left\{\frac{1}{8N}\left(1\mp\varepsilon\right)^2, \frac{1}{2}\left(1-\varepsilon^2\right)\right\}\right), \tag{S.72}$$

$$K_{dG}^\phi(\Delta) = K_{dG}^\phi(0) = \gamma_\Delta^\phi\left(\left\{\frac{1}{2N}, 0\right\}\right), \tag{S.73}$$

$$K_{d_id_j}^\phi(\Delta) = K_{d_id_j}^\phi(0) = 0. \tag{S.74}$$

When the detuning is large, multi phonon dephasing processes are suppressed by $1-\varepsilon^2$. Moreover, Eq. (S.71) shows that non-resonance breaks the equality of the polariton dephasing rates such that $K_{+G}^\phi(\Delta) > K_{-G}^\phi(\Delta)$. However, the symmetry breaking occurs in the single phonon dephasing processes, which are not the leading order contribution, and so this effect may be too small to observe even for a large detuning.

D. Lamb shifts

We will write the Lamb shifts in terms of a generalized Lamb shift function,

$$S_\Delta(\nu,\{a,b,c\}) = a\ S_1^v(\nu) + b\ S_{>1}^{\mathrm{even}}(\nu) + c\ S_{>1}^{\mathrm{odd}}(\nu) \tag{S.75}$$

where $S_1^v(\nu)$ describes single phonon processes and is given in Eq. (76). The remaining functions are $S_{>1}^{\text{even}}(\nu) = \text{Im}[\Gamma_{>1}^{\text{even}}(\nu)]$ and $S_{>1}^{\text{odd}}(\nu) = \text{Im}[\Gamma_{>1}^{\text{odd}}(\nu)]$ where $\Gamma_{>1}^{\text{even}}(\nu)$ and $\Gamma_{>1}^{\text{odd}}(\nu)$ are given in Eq. (S.61). In terms of Eq. (S.75) the Lamb shift induced by transitions between polaritons is

$$\Lambda^t_{\pm\to\mp}(\Delta) = \frac{1}{4N} S_\Delta(\pm\theta, \{1-\varepsilon^2, 2\varepsilon^2, 2\}), \tag{S.76}$$

and the shift induced by transitions from polaritons to dark states is

$$\Lambda^t_{\pm\to d}(\Delta) = \frac{1}{2N} S_\Delta \left(\pm\frac{\theta\mp\Delta}{2}, \{1\pm\varepsilon, 1\mp\varepsilon, 1\mp\varepsilon\}\right). \tag{S.77}$$

The superscript 't' denotes that this arises from real transitions. The Lamb shifts induced by transitions from dark states to polaritons are obtained from Eq. (S.77) by inverting the sign in the first argument of the function on the right-hand-side. The Lamb shift induced by transitions between the degenerate dark states is equal to the resonant value, $\Lambda^t_{d\to d'\neq d} = S_1(0)/N$. Notice that Lamb shifts induced by transitions have the same dependencies on detuning as the transitions which generate them, described in Eqs. (S.63) and (S.64).

Lastly, we describe the Lamb shifts induced by virtual self transitions through the generalized function

$$S_\Delta^\phi(\{a,b\}) = a\ S_1^v(0) + b\ S_{>1}^\phi(0), \tag{S.78}$$

where $S_{>1}^\phi(0)$ is the multi phonon contribution defined through Eq. (64). One finds the following virtual self Lamb shifts,

$$\Lambda^s_{\pm\to\pm}(\Delta) = \frac{1}{4N} S_\Delta^\phi\left(\{1\pm\epsilon, 1-\epsilon^2\}\right), \tag{S.79}$$

$$\Lambda^s_{d\to d}(\Delta) = \Lambda^s_{d\to d}(0) = \frac{1}{N} S_\Delta^\phi\left(\{1,0\}\right). \tag{S.80}$$

(S.81)

Combining the Lamb shifts from virtual and real transitions, the total shift of each eigenstate is

$$\Lambda_\pm(\Delta) = \Lambda_{\pm\to\mp}(\Delta) + (N-1)\Lambda_{\pm\to d}(\Delta) + \Lambda_{\pm\to\pm}(\Delta), \tag{S.82}$$

$$\Lambda_d(\Delta) = \Lambda_{d\to+}(\Delta) + \Lambda_{d\to-}(\Delta) + \frac{N-2}{N} S_1(0) + \Lambda_{d\to d}(\Delta), \tag{S.83}$$

where each Lamb shift is a summation of the contribution from real and virtual transitions, e.g., $\Lambda_{+\to-}(\Delta) = \Lambda^t_{+\to-}(\Delta) + \Lambda^s_{+\to-}(\Delta)$.

REFERENCES

[1] Quach, J. Q.; McGhee, K. E.; Ganzer, L.; Rouse, D. M.; Lovett, B. W.; Gauger, E. M.; Keeling, J.; Cerullo, G.; Lidzey, D. G.; Virgili, T. Superabsorption in an organic microcavity: Toward a quantum battery. *Science advances* **2022**, *8*, eabk3160.

[2] del Pino, J.; Feist, J.; Garcia-Vidal, F. J. Quantum theory of collective strong coupling of molecular vibrations with a microcavity mode. *New Journal of Physics* **2015**, *17*, 053040.

[3] Ficek, Z.; Swain, S. *Quantum interference and coherence: theory and experiments*; Springer Science & Business Media, 2005; Vol. 100.

[4] Nazir, A.; McCutcheon, D. P. Modelling exciton–phonon interactions in optically driven quantum dots. *Journal of Physics: Condensed Matter* **2016**, *28*, 103002.

[5] McCutcheon, D. P. Optical signatures of non-Markovian behavior in open quantum systems. *Physical Review A* **2016**, *93*, 022119.

[6] Iles-Smith, J.; Diba, O.; Nazir, A. Capturing non-Markovian polaron dressing with the master equation formalism. *arXiv preprint arXiv:2407.10744* **2024**,

[7] Breuer, H.-P.; Petruccione, F. *The theory of open quantum systems*; Oxford University Press, USA, 2002.

[8] Pollock, F. A.; McCutcheon, D. P.; Lovett, B. W.; Gauger, E. M.; Nazir, A. A multi-site variational master equation approach to dissipative energy transfer. *New Journal of Physics* **2013**, *15*, 075018.

[9] Shalabney, A.; George, J.; Hutchison, J. a.; Pupillo, G.; Genet, C.; Ebbesen, T. W. Coherent coupling of molecular resonators with a microcavity mode. *Nature communications* **2015**, *6*, 5981.
